# Supplementary material for: Multicentre double-blind randomised placebo-controlled trial evaluating the efficacy of the meningococcal B vaccine, 4CMenB (Bexsero), against Neisseria gonorrhoeae infection in men who have sex with men: the GoGoVax study protocol
Source: BMJ Open. 2024 Apr 16;14(4):e081675. doi: 10.1136/bmjopen-2023-081675 (PMC11029339; doi:10.1136/bmjopen-2023-081675)
Supplement: Author's manuscript [file bmjopen-2023-081675.draft_revisions.pdf]

BMJ Open is committed to open peer review. As part of this commitment we make the peer review history of every article we publish publicly available.

When an article is published we post the peer reviewers' comments and the authors' responses online. We also post the versions of the paper that were used during peer review. These are the versions that the peer review comments apply to.

The versions of the paper that follow are the versions that were submitted during the peer review process. They are not the versions of record or the final published versions. They should not be cited or distributed as the published version of this manuscript.

BMJ Open is an open access journal and the full, final, typeset and author-corrected version of record of the manuscript is available on our site with no access controls, subscription charges or pay-per-view fees (http://bmjopen.bmj.com).

If you have any questions on BMJ Open's open peer review process please email info.bmjopen@bmj.com

BMJ Open

A multicentre double-blind randomised placebo-controlled trial evaluating the efficacy of the meningococcal B vaccine, 4CMenB (Bexsero), against Neisseria gonorrhoeae infection in men who have sex with men: the GoGoVax study Protocol

Journal:	*BMJ Open*
Manuscript ID	bmjopen-2023-081675
Article Type:	Protocol
Date Submitted by the Author:	03-Nov-2023
Complete List of Authors:	Seib , KL; Griffith University Institute for Glycomics, ; Griffith University<br>Donovan, Basil; University of New South Wales, The Kirby Institute<br>Thng, Caroline; Queensland Health, Gold Coast Sexual Health<br>Lewis, David ; Western Sydney Sexual Health Centre<br>McNulty, Anna; Sydney Sexual Health Centre,<br>Fairley, Christopher; Melbourne Sexual Health Centre,<br>Yeung, Barbara; The Kirby Institute, UNSW<br>Jin, Fengyi; The Kirby Institute, UNSW,<br>Fraser, Doug; The Kirby Institute, UNSW<br>Bavinton , BR; The Kirby Institute<br>Law, Matthew; University of New South Wales,<br>Chen, Marcus; Melbourne Sexual Health Centre, The Alfred Hospital<br>Whiley, David; The University of Queensland, Queensland Children's Medical Research Institute<br>Mackie, Brent; ACON<br>Jennings, Michael; Griffith University, Institute for Glycomics<br>Jennison, Amy; Public Health Microbiology, Queensland Health Forensic and Scientific Services,<br>Lahra, Monica M; New South Wales Health Pathology<br>Grulich, Andrew; The Kirby Institute, ;
Keywords:	Vaccination, Clinical Trial, IMMUNOLOGY, MICROBIOLOGY, Sexually Transmitted Disease

SCHOLARONE™ Manuscripts

A multicentre double-blind randomised placebo-controlled trial evaluating the efficacy of the meningococcal B vaccine, 4CMenB (Bexsero), against *Neisseria gonorrhoeae* infection in men who have sex with men: the GoGoVax study Protocol

Kate L Seib [1*], Basil Donovan [2], Caroline Thng [3], David A Lewis [4,5], Anna McNulty [6,7], Christopher K Fairley [8,9], Barbara Yeung [2], Fengyi Jin [2], Doug Fraser [2], Benjamin R Bavinton [2], Matthew Law [2], Marcus Y Chen [8,9], Eric PF Chow [8,9], David M Whiley [10], Brent Mackie [11], Michael P Jennings [1], Amy V Jennison [12], Monica M Lahra [13], Andrew E Grulich [2].

[1] Institute for Glycomics, Griffith University, Gold Coast, QLD, Australia
[2] The Kirby Institute, University of New South Wales, Sydney, NSW, Australia
[3] Gold Coast Sexual Health, Southport, QLD, Australia
[4] Western Sydney Sexual Health Centre, Sydney, NSW, Australia
[5] Sydney Medical School-Westmead, Faculty of Medicine and Health and Sydney Infectious Diseases Institute, University of Sydney, Sydney, NSW, Australiasdfdfdfd
[6] Sydney Sexual Health Centre, Sydney, NSW, Australia
[7] School of Population Health, UNSW Sydney, Sydney, Australia.
[8] Melbourne Sexual Health Centre, Melbourne, VIC, Australia
[9] Central Clinical School, Faculty of Medicine, Nursing and Health Sciences, Monash University, Melbourne, VIC, Australia
[10] UQ Centre for Clinical Research, Faculty of Medicine, The University of Queensland, Brisbane, QLD, Australia
[11] ACON Health, NSW, Australia
[12] Public Health Microbiology, Queensland Health Forensic and Scientific Services, Brisbane, QLD, Australia
[13] WHO Collaborating Centre for STI and AMR, NSW Health Pathology Microbiology. The Prince of Wales Hospital, Sydney, NSW, Australia

*Correspondence: Kate L Seib, k.seib@griffith.edu.au

For peer review only - http://bmjopen.bmj.com/site/about/guidelines.xhtml

ABSTRACT

Introduction Gonorrhoea, the sexually transmissible infection caused by *Neisseria gonorrhoeae*, has a substantial impact on sexual and reproductive health globally with an estimated 82 million new infections each year worldwide. *N. gonorrhoeae* antimicrobial resistance continues to escalate, and disease control is largely reliant on effective therapy as there is no proven effective gonococcal vaccine available. However, there is increasing evidence from observational cohort studies that the serogroup B meningococcal vaccine 4CMenB (trade name Bexsero®), licenced to prevent invasive disease caused by *Neisseria meningitidis*, may provide cross-protection against the closely related bacterium *N. gonorrhoeae.* This study will evaluate the efficacy of 4CMenB against *N. gonorrhoeae* infection in men (cis and trans), trans women and non-binary people who have sex with men (hereafter referred to as GBM+).

Methods and analysis This is a double-blind, randomised placebo-controlled trial in GBM+, either HIV-negative on pre-exposure prophylaxis (PrEP) against HIV or living with HIV (CD4 count >350 cells/mm^3), who have had a diagnosis of gonorrhoea or infectious syphilis in the last 18 months (a key characteristic associated with a high risk of *N. gonorrhoeae* infection). Participants are randomised 1:1 to receive two doses of 4CMenB or placebo 3 months apart. Participants have 3-monthly visits over 24 months, which include testing for *N. gonorrhoeae* and other sexually transmissible infections, collection of demographics, sexual behaviour risks and antibiotic use, and collection of research samples for analysis of *N. gonorrhoeae*-specific systemic and mucosal immune responses. The primary outcome is the incidence of the first episode of *N. gonorrhoeae* infection, as determined by nucleic acid amplification tests (NAATs), post month 4. Additional outcomes consider the incidence of symptomatic or asymptomatic *N. gonorrhoeae* infection at different anatomical sites (i.e., urogenital, anorectum, or oropharynx), incidence by *N. gonorrhoeae* genotype and antimicrobial resistance phenotype, and level and functional activity of *N. gonorrhoeae*-specific antibodies.

Ethics and dissemination Ethical approval was obtained from the St Vincent's Hospital Human Research Ethics Committee (HREC), St Vincent's Hospital Sydney, NSW, Australia (ref: 2020/ETH01084). Results will be disseminated in peer-reviewed journals and via presentation at national and international conferences.

Trial registration ClinicalTrials.gov Identifier NCT04415424.

Keywords
Gonorrhoea, *Neisseria gonorrhoeae,* sexually transmissible infection (STI), men who have sex with men (MSM), gay and bisexual men (GBM), meningococcal vaccine, 4CMenB, Bexsero®.

Strengths and limitations of this study

- This is the first double-blind randomised placebo-controlled trial to evaluate the efficacy of the four-component meningitis B vaccine (4CMenB) against *N. gonorrhoeae* infection in men who have sex with men and will provide information regarding vaccine efficacy against symptomatic and asymptomatic infection at different anatomic sites.
- Antimicrobial resistance (AMR) testing and genome sequencing of *N. gonorrhoeae* positive samples will provide information regarding the circulating strains in the study population, and the vaccine efficacy against circulating strains including those with differing AMR profiles.
- Immunological analysis of samples from the trial will provide insight into the *N. gonorrhoeae* specific immune response following 4CMenB vaccination.
- Limitations of the study are that it does not include cisgender women, immunosuppressed individuals living with HIV (CD4 count ≤350 cells/mm^3), or participants with a low risk of *N. gonorrhoeae* infection.

For peer review only - http://bmjopen.bmj.com/site/about/guidelines.xhtml

INTRODUCTION

Gonorrhoea is a global public health concern due to its prevalence, the severe sequelae it can cause if left untreated, and the increasing difficulty in treating multi-drug resistant strains of *Neisseria gonorrhoeae.*[1 2] There are estimated to be more than 82 million new cases of *N. gonorrhoeae* infections annually worldwide,[3] and the incidence is rising. Between 2012 and 2019 there was a 127% increase in notified gonorrhoea cases in Australia,[4] and a 74% increase in the USA.[5] In Australia, the majority of gonococcal infections are in are in gay, bisexual and other men who have sex with men (GBM).[6] An Australian state-wide implementation project of pre-exposure prophylaxis (PrEP) against HIV infection in GBM documented a gonorrhoea incidence of 39.0 per 100-person years (PY), with infected sites including the urethra (7.3/100 PY), pharynx (19.7/100 PY), and anus (22.6/100 PY).[7] Analysis from sexual health clinics across Australia indicated that HIV-positive GBM had a gonorrhoea incidence of 29.1/100 PY.[8]

The outcomes of *N. gonorrhoeae* infection vary by site of infection and by sex.[1] Oropharyngeal and anorectal infections are typically asymptomatic. Symptomatic gonococcal infection typically presents as urethritis in males and cervicitis in females, although up to 10% of male and ~30-80% of female genital tract infections are asymptomatic.[3] Undiagnosed or untreated urogenital tract infections can lead to severe sequelae, including urethral stricture, urogenital tract abscesses, epididymo-orchitis and infertility in males, and pelvic inflammatory disease, adverse pregnancy outcomes and infertility in females.[3] *N. gonorrhoeae* occasionally disseminates, resulting in sepsis, septic arthritis, perihepatitis, endocarditis and/or meningitis. Infection with *N. gonorrhoeae* may also increase the risk of acquiring and transmitting HIV.[1]

N. gonorrhoeae AMR is of utmost concern, particularly with emerging resistance to ceftriaxone. In 2018 extensively drug-resistant (and ceftriaxone-resistant) *N. gonorrhoeae* were identified in Australia, resistant to all commonly used antibiotics, and very recently further isolates were reported in the UK, Austria, Cambodia, and France.[9-16] As such, *N. gonorrhoeae* has been prioritised as an urgent public health threat for which new preventive measures are needed, such as a vaccine.[17-19] Unfortunately, gonococcal vaccine development has been unsuccessful to date and there are large knowledge gaps regarding what constitutes a protective immune response for *N. gonorrhoeae.*[1] However, there is increasing evidence from observational cohort and case-control studies that outer membrane vesicle (OMV)-based *Neisseria meningitidis* (meningococcal) serogroup B (MenB) vaccines may provide cross-protection against gonorrhoea.[20-23] *N. gonorrhoeae* and *N. meningitidis* are closely related bacteria, with conservation of the majority of genes [24] and thus also conservation of outer membrane proteins that could be targeted by vaccines. Observational studies have detected a decrease in incidence of *N. gonorrhoeae* infection following vaccination with the outer membrane vesicle (OMV) meningococcal vaccines MeNZB [20] or 4CMenB [21-23] with vaccine effectiveness estimated to be approximately 30-50%. The OMVs in these vaccines are structures that are released from the outer membrane of Gram-negative bacteria, and contain phospholipids, lipooligosaccharide and a complex mix of proteins. The broader spectrum four-component serogroup B vaccine, 4CMenB (marketed as Bexsero®) contains the MeNZB OMV antigens plus an additional three recombinant antigens (NHBA-GNA1030 and fHbp-GNA2091 fusions, and NadA).[25] There are homologues of 20 of the 22 major OMV vaccine proteins present in *N gonorrhoeae*, as well as the NHBA antigen.[26] Therefore, there are several vaccine antigens that may be responsible for inducing an immune response that cross reacts with *N. gonorrhoeae.* The observational study findings with MenB OMV-based vaccines are the first time that any vaccine has been associated with protection against gonorrhoea. However, data from a randomised control trial is required to confirm the efficacy of 4CMenB to protect against *N. gonorrhoeae* infection.

STUDY AIMS AND OBJECTIVES

This study aims to evaluate the efficacy of 4CMenB in the prevention of *N. gonorrhoeae* infection in men (cis and trans), trans women, trans men, and non-binary people who have sex with men (hereafter referred to as GBM+). The primary outcome is the incidence of the first episode of *N. gonorrhoeae*

For peer review only - http://bmjopen.bmj.com/site/about/guidelines.xhtml

infection, as determined by nucleic acid amplification tests (NAATs), post month 4 (i.e., after 4 weeks post dose two). Secondary outcomes are the incidence of symptomatic or asymptomatic *N. gonorrhoeae* infection at different anatomical sites (i.e., urogenital, anorectum, or oropharynx), incidence by *N. gonorrhoeae* genotype and antimicrobial resistance phenotype, and level and functional activity of any induced *N. gonorrhoeae*-specific antibodies. Primary, secondary and immunogenicity objectives and endpoint measures are outlined in Table 1.

METHODS AND ANALYSIS

Study design and setting

The study is a double-blind, randomised placebo-controlled trial of a two-dose schedule of 4CMenB to prevent urethral, oropharyngeal, and anal *N. gonorrhoeae* infection in GBM+, who are either taking HIV PrEP or postexposure prophylaxis (PEP), are planning to start PrEP after the cessation of PEP, or are living with HIV with a CD4 count >350 cells/mm^3. Participants are randomised 1:1 to receive two doses of 4CMenB or saline placebo 3 months apart. Participants are required to attend 3-monthly follow-up visits over 24 months. At each visit there is testing for *N. gonorrhoeae* and other sexually transmissible infections including HIV, collection of blood and oral mucosal exudate samples for analysis of *N. gonorrhoeae*-specific immune responses, and collection of demographics, sexual behaviour risks, and antibiotic use. An overview of the study design is shown in Figure 1 and a summary of the study visits is shown in Table 2.

The study is being conducted at five public sexual health clinics and two private clinics in New South Wales, Victoria and Queensland, Australia: the Sydney Sexual Health Centre (SSHC), the Western Sydney Sexual Health Centre (WSSHC), the Melbourne Sexual Health Centre (MSHC), the Gold Coast Sexual Health Service (GCSHS), Royal Prince Alfred Sexual Health (RPASH) Sydney, Taylor Square Private Clinic (TSPC) Sydney, and Prahran Market Clinic (PMC) Melbourne.

Study population, recruitment, and eligibility criteria

The participant population for this trial is GBM+ taking PrEP/PEP or living with HIV (CD4 count >350 cells/mm^3), who have had a diagnosis of gonorrhoea or infectious syphilis in the last 18 months (a key characteristic associated with greater gonorrhoea incidence[7]). This population is recommended under Australian guidelines to attend clinics 3-monthly for comprehensive sexual health screening, including NAAT-based screening of urogenital, oropharyngeal, and anal specimens for *N. gonorrhoeae* infection.[6 27]

Potentially eligible participants were identified by clinicians at each clinic based on the inclusion and exclusion criteria (see Box 1) and were referred to the study clinicians or study nurses who explained the study, checked eligibility, and obtained informed consent. Potentially eligible participants were also referred to the participating clinics by other public sexual health centres, public health clinics, general practices, and state-based lesbian, gay, bisexual, and transgender community organisations, such as ACON and Positive Life NSW in NSW, Thorne Harbour Health and Living Positive Victoria in Victoria, and the Queensland Council for LGBTI Health and Queensland Positive People.

Study processes

Randomisation and blinding

Once recruited, participants were randomly assigned (1:1) and stratified by clinical sites via a web-based randomisation system, to receive either 4CMenB or saline placebo. Participants, their clinicians (site investigators/doctors, site coordinators/nurses) and study researchers assessing the outcomes were blinded to the assignment. The site pharmacists and drug manager who dispensed the study treatment, and the vaccinators who administered the study treatment were unblinded to the treatment allocation.

Intervention

A pre-vaccination checklist was completed prior to administration of intervention to ensure safety of

For peer review only

participants. At the month 0 (Baseline) and month 3 visits participants received either a dose of 0.5 ml 4CMenB or a dose of 0.5 ml placebo (150 mmol of sodium chloride / 0.9% saline solution) by intramuscular injection. This vaccine schedule is consistent with 4CMenB's indication for immunisation against invasive disease caused by *N. meningitidis* group B in people >2 years of age (two doses with an interval of ≥1 month) [28]. 4CMenB is a licensed vaccine in Australia with well documented safety profile but it is not approved by the Therapeutic Good Administration (TGA) for the prevention of gonorrhoea. Therefore, the trial is being conducted under the Clinical Trial Notification (CTN) scheme. At the completion of study, participants who were randomised to the placebo arm will be given the opportunity to receive the 4CMenB vaccine.

Sample and data collection

The study assessments, samples, and data collected at each 3-monthly visits over 24 months are outlined in Figure 1 and Table 2. At each 3-monthly visit, study participants have routine specimens collected as part of their PrEP or HIV clinical care as per the Australian STI Management Guidelines.[29] These specimens include urogenital, oropharyngeal and anorectal specimens for screening for *N. gonorrhoeae* and *Chlamydia trachomatis* by nucleic acid amplification test (NAAT), and blood for screening for HIV and syphilis. At each visit, data are also recorded regarding symptoms that may be related to gonorrhoea including urethritis, proctitis, epididymitis, and cervicitis/vaginitis. Unscheduled visits to the participating sites or external healthcare providers, at which gonorrhoea is diagnosed by a NAAT, are also recorded.

Participants complete a study questionnaire at baseline to collect demographics and sexual risk behaviour. A questionnaire is also completed at months 12 and 24 to collect ongoing sexual risk behaviour. At each visit, recent antibiotic use, and history of meningococcal B vaccination within the last 3 months is also recorded.

Research specimens (blood and oral mucosal transudates) are collected at baseline, and at Months 0, 6, 12, and 24 at selected study sites to investigate vaccine-induced antibodies via assays including enzyme-linked immunosorbent assays (ELISA), serum bactericidal activity (SBA), opsonophagocytic killing (OPA), and antigen function blocking assays.[26 30 31]. Culture isolates collected as part of the routine care for participants with *N. gonorrhoeae* infection will be used to conduct phenotypic antimicrobial resistance testing [32 33] and whole genome sequence analysis.[34 35] Testing will be performed at the WHO Collaborating Centre for STIs and AMR at the Prince of Wales Hospital in Sydney, the Queensland Forensic and Scientific Services at Queensland Health in Brisbane, and/or at the Microbiological Diagnostic Unit in Melbourne.

Safety reporting

4CMenB is a licensed vaccine with a well-documented safety and tolerability profile. [36] Therefore, safety monitoring is focused on serious adverse events (SAEs). All SAEs will be reported within 24 hours to the Study Sponsor and the Project Medical Officer (at the Kirby Institute) and to the HREC annually. The Sponsor will notify the TGA, HREC and site principal investigators of all significant events that can adversely affect the safety of participants.

Data management

Source documents include, but are not limited to participant medical records, laboratory reports, participant progress notes, pharmacy records and any other reports or records of procedures performed in accordance with the protocol. Study data are collected using the Medrio Electronic Data Capture system (Medrio Inc., San Francisco, CA, US) with a web-enabled password protected platform. Each participant is assigned a unique Participant Identification Number (PIN) that is documented in the participant's medical record and all study documents including the dispensing records. Following each participant visit the designated site staff complete the visit-specific electronic case report form (eCRF) as soon as possible after the completion of the visit. Participants also provide

For peer review only - http://bmjopen.bmj.com/site/about/guidelines.xhtml

consent for the Project Team to acquire any other information and results from other health services for the purpose of the research via linkage with the Australian Collaboration for Coordinated Enhanced Sentinel Surveillance (ACCESS) system.[37] Data from ACCESS will enable long-term follow-up after the completion of the study even if a participant moves to another clinic.

Sample size

The primary endpoint of the study will be incidence of first episode of *N. gonorrhoeae* infection (urogenital, anal, or oropharyngeal) as determined by NAAT testing, per 100 PY, post month 4. Assuming a vaccine efficacy of 30% (hazard ratio=0.7; as predicted for MeNZB™ [20]), and a conservative *N. gonorrhoeae* incidence of 25 per 100 PY (based on recent Australian data for GBM on PrEP (39 per 100 PY)[7] and in HIV-positive GBM (29 per 100 PY)[8]), 1:1 randomisation, a 12-month recruitment period, and 24-month follow-up, and 10% lost to follow-up, a total of 730 participants (365 randomised to each arm) would need to be recruited to have 80% power to demonstrate vaccine efficacy (2-alpha=5%).

Statistical analysis

The primary efficacy endpoint is the incidence of first episode of *N. gonorrhoeae* infection detected by a NAAT, post month 4. A secondary endpoint will include the incidence of all episodes of *N. gonorrhoeae* infection detected by a NAAT. All episodes of chlamydial infection diagnosed during the study period will also be captured for the proposed additional analyses to compare the incidence of chlamydial infection between the vaccine arm and the placebo arm.

Analysis populations

The primary analysis of efficacy will be per-protocol, including study participants who received two vaccine or placebo doses, have two or more follow-up visits following the second dose, and do not fail on any exclusion criterion pertinent to the assessment of efficacy. In this analysis, only *N. gonorrhoeae* infections 4 weeks post dose 2 (i.e., after Month 4) will be considered. A modified intent-to-treat (MITT) analysis will be performed as a secondary analysis including all participants (including general protocol violators) who receive at least 1 dose of vaccine or placebo and have at least 6 months of study follow-up after the first dose. For these analyses, any efficacy against cases occurring after Day 1 visit will be counted.

Statistical Methods

Vaccine efficacy (VE) will be defined as:

$$VE = 100\%\{1- (r_v/r_p)\}$$

where r_v is the incidence rate among vaccine recipients and r_p is the incidence rate among placebo recipients. Each efficacy hypothesis will be tested by constructing a two-sided 95% confidence interval for VE using Poisson regression models, accommodating repeated measures for the secondary endpoint.

Patient and public involvement

Representatives from the community groups ACON (an LGBTQ+ health organisation) and Positive Life NSW (a community organisation for people living with HIV) have been involved in the research since the initial funding application and have participated in monthly/2-monthly management team meetings since the start of trial planning. They provided input on the protocol, study information and recruitment materials. These community partners will assist in dissemination of study results to participants and the community via their social media platforms.

DISCUSSION

In light of increasing gonorrhoea incidence and escalating antimicrobial resistance, there is an urgent need for new gonorrhoea prevention strategies. The World Health Organization (WHO) has set a

For peer review only - http://bmjopen.bmj.com/site/about/guidelines.xhtml

target for reducing gonorrhoea incidence by 90% by 2030 and highlighted the need for an effective vaccine to help achieve this goal[38]. GoGoVax is the first double-blind, randomised placebo-controlled trial to investigate the efficacy of the meningococcal 4CMenB vaccine against *N. gonorrhoeae* infection in GBM+. This trial will provide critical scientific evidence to inform potential indication expansion of 4CMenB to include gonorrhoea, which could lead to a substantial reduction in *N. gonorrhoeae* incidence worldwide. Observational evidence to date suggests that 4CMenB may have a vaccine effectiveness against *N. gonorrhoeae* of approximately 30-50%.[21-23] Mathematical modelling in an MSM population indicates that a gonococcal vaccine with 25-50% efficacy and 2-years duration of protection could reduce gonorrhoea prevalence by 31-62% in 2 years if 30% of MSM presenting for STI screening are vaccinated per visit.[39]

Our study addresses several questions in the field regarding overall protection against *N. gonorrhoeae* infection provided by 4CMenB, but also regarding vaccine efficacy against symptomatic and asymptomatic infection at genital and extragenital sites of infection, efficacy against diverse strains, as well as duration and potential mechanisms of vaccine-mediated protection. The link between symptoms and *N. gonorrhoeae* load and transmission is unclear [40-42] and it is unknown if vaccine efficacy will differ depending on the presence or absence of symptoms. Vaccine efficacy may also differ by anatomical site of infection. This has been seen for antibiotic treatment of gonorrhoea, with antibiotics being less effective against oropharyngeal gonorrhoea than for urethral and rectal infections.[43 44] Although 4CMenB is able to prevent invasive meningococcal disease, it is not believed to result in a clinically significant reduction in nasopharyngeal carriage of *N. meningitidis*. [45-47] However, unlike *N. meningitidis*, *N. gonorrhoeae* does not have a polysaccharide capsule which may alter its susceptibility to 4CMenB vaccine-induced immune responses in the pharynx. Given the diversity of *N. gonorrhoeae*, the antimicrobial resistance testing and genome sequencing of *N. gonorrhoeae* positive samples will provide information regarding the circulating strains in the population to enable evaluation of vaccine efficacy against circulating strains, including of differing AMR profiles. Linkage between our study and the Australian ACCESS surveillance system[37] will allow assessment of durability of any preventive effect of 4CMenB beyond the duration of the trial. Immunological analysis of samples from the trial will provide insight into the *N. gonorrhoeae*-specific immune response following 4CMenB vaccination. Immunological correlates of protection for *N. gonorrhoeae* have not yet been identified, [48 49] and insight from this trial will facilitate future vaccine development and evaluation.

This trial is targeted at the people at highest risk. Men who have sex with men and people living with HIV are key populations at higher risk for gonococcal infection [48] and Australian GBM+ are diagnosed with some of the highest gonorrhoea incidence rates ever described.[7 8] As such, it is essential to understand whether 4CMenB is able to prevent or reduce *N. gonorrhoeae* in this population. However, it should be noted that we did not enrol immunosuppressed men living with HIV (CD4 count <350 cells/mm^3) in order to optimise the immune response to vaccine in participants. Another important limitation of our study is the lack of inclusion of cisgender women. Gonorrhoea causes a significant burden of disease in females, and it is possible that past *N. gonorrhoeae* infection may impact vaccine-induced immune responses. Additional 4CMenB efficacy trials currently underway in different settings that include female participants (e.g., clinicaltrials.gov NCT04350138) will hopefully address some of these limitations and complement findings from our study.

Due for completion in 2025, the GoGoVax study will inform future gonorrhoea prevention strategies. If 4CMenB is proven to be effective against *N. gonorrhoeae*, this could facilitate changes in gonorrhoea prevention strategies that could improve sexual and reproductive health worldwide.

ETHICS AND DISSEMINATION

For peer review only

For peer review only - http://bmjopen.bmj.com/site/about/guidelines.xhtml

The current study protocol (version 5, 24 June 2022) informed consent forms, and all recruitment materials have been reviewed and approved by St Vincent's Hospital Human Research Ethics Committee, St Vincent's Hospital Sydney, NSW, Australia (ref: 2020/ETH01084). The study results will be published in international peer-reviewed journals, presented at national and international conferences, and provided to study participants and the public via social, television, radio and/or print media.

Acknowledgements

We would like to acknowledge the GoGoVax Protocol Steering Committee members and the site investigators and coordinators.

Protocol Steering Committee members:

Prof Kate Seib, Prof Basil Donovan, Scientia Prof Andrew Grulich, Prof Christopher Fairley, Prof David Lewis, A/Prof Anna McNulty, Dr Caroline Thng, A/Prof Mark Polizzotto, A/Prof David Whiley, Prof Matthew Law, A/Prof Eric Chow, Dr Rob Finlayson, Dr Mark O'Reilly, Dr Nick Comninos, Dr Rick Varma, Dr Norman Roth, Dr Beng Eu, Prof David Templeton, Dr Amy Jennison, Mr Brent Mackie, Mr Andrew Heslop, Ms Jane Costello, Dr Christine Selvey, Prof Deborah Williamson, Prof John Kaldor, A/Prof Marcus Chen, Prof Michael Jennings, Prof Monica Lahra, Prof Rebecca Guy, Dr Fengyi Jin, Dr Benjamin Bavinton, Ms Barbara Yeung, Mr Doug Fraser, Ms. Michelle Burns.

Site investigators and coordinators:

Sydney Sexual Health Centre - A/Prof Anna McNulty, Dr Rick Varma, Mr. Paul Robinson, Mr. Anik Ray, Ms. Alison Jenkins.

Western Sydney Sexual Health Centre - Prof David Lewis, Dr Nick Comninos, Dr Rohan Bopage, Ms. Melissa Power, Mr. Pradeep Kumar.

Melbourne Sexual Health Centre - Prof Christopher Fairley, A/Prof Eric Chow, A/Prof Marcus Chen, Ms. Kate Maddaford, Mr. Finn Mercury.

Gold Coast Sexual Health Services - Dr Caroline Thng, Ms. Catherine Donald, Helen McEvoy.

RPA Sexual Health - Prof David Templeton, Dr David Atefi, Dr Rachel Burdon, Dr Lara Whitbourne, Ms. Linda Garton, Ms. Ashlee Jackson.

Taylor Square Private Clinic - Dr Rob Finlayson, Dr Mark O'Reilly, Ms. Piper Calleia, Mr. Kiran Muthukrishnan.

Prahran Market Clinic - Dr Norman Roth, Dr Beng Eu, Ms. Helen Lau.

Contributors

KLS, BD and AEG designed the study and wrote the initial manuscript. All authors contributed to study design, protocol development, and manuscript review, and approved the final version of the manuscript for submission.

Funding

This work was supported by the Australian National and Medical Health Council (NHMRC) grant number 1182443. Additional support was provided by Griffith University (Administrative Institute) and the Kirby Institute UNSW Sydney (Study Sponsor). Vaccine was provided by GlaxoSmithKline (GSK) Australia Pty Ltd. The funding bodies, study sponsor and GSK had no role in the design of the study. KLS acknowledges supported from the NHMRC (grants 2017383 and 2002182). EPFC is supported by an NHMRC Emerging Leadership Investigator Grant (GNT1172873).

Competing interests

None

For peer review only

For peer review only - http://bmjopen.bmj.com/site/about/guidelines.xhtml

Ethics approval St Vincent's Hospital Human Research Ethics Committee (HREC/2020/ETH01084).

Patient consent for publication Not required.
Provenance and peer review Not commissioned; externally peer reviewed.

References

1. Edwards JL, Jennings MP, Apicella MA, et al. Is gonococcal disease preventable? The importance of understanding immunity and pathogenesis in vaccine development. *Crit Rev Microbiol* 2016;42(6):928-41. doi: 10.3109/1040841X.2015.1105782 [published Online First: 2016/01/26]
2. Unemo M, Bradshaw CS, Hocking JS, et al. Sexually transmitted infections: challenges ahead. *The Lancet Infectious diseases* 2017;17(8):e235-e79. doi: 10.1016/S1473-3099(17)30310-9 [published Online First: 2017/07/14]
3. WHO. Global incidence and prevalence of selected curable sexually transmitted infections – 2008 2012 [Available from: http://www.who.int/reproductivehealth/publications/rtis/stisestimates/en/ accessed 7 March 2015.
4. Kirby Institute. HIV, viral hepatitis and sexually transmissible infections in Australia: annual surveillance report 2022. The Kirby Institute, UNSW Sydney, 2022.
5. CDC. Sexually Transmitted Disease Surveillance 2021. *https://www.cdc.gov/std/statistics/* 2021.
6. ASHM. The Australian STI Management Guidelines. *http://wwwstiguidelinesorgau/* 2019.
7. Traeger MW, Cornelisse VJ, Asselin J, et al. Association of HIV Preexposure Prophylaxis With Incidence of Sexually Transmitted Infections Among Individuals at High Risk of HIV Infection. *JAMA* 2019;321(14):1380-90. doi: 10.1001/jama.2019.2947 [published Online First: 2019/04/10]
8. Callander D, Guy R, Fairley CK, et al. Gonorrhoea gone wild: rising incidence of gonorrhoea and associated risk factors among gay and bisexual men attending Australian sexual health clinics. *Sex Health* 2019;16(5):457-63. doi: 10.1071/SH18097 [published Online First: 2018/11/10]
9. Eyre DW, Sanderson ND, Lord E, et al. Gonorrhoea treatment failure caused by a Neisseria gonorrhoeae strain with combined ceftriaxone and high-level azithromycin resistance, England, February 2018. *Euro Surveill* 2018;23(27) doi: 10.2807/1560-7917.ES.2018.23.27.1800323 [published Online First: 2018/07/12]
10. ECDC. Extensively drug-resistant (XDR) Neisseria gonorrhoeae in the United Kingdom and Australia. *European Centre for Disease Prevention and Control Stockholm https://ecdceuropaeu* 2018
11. Whiley DM, Jennison A, Pearson J, et al. Genetic characterisation of Neisseria gonorrhoeae resistant to both ceftriaxone and azithromycin. *Lancet Infect Dis* 2018;18(7):717-18. doi: 10.1016/S1473-3099(18)30340-2 [published Online First: 2018/07/07]
12. Maubaret C, Camelena F, Mrimeche M, et al. Two cases of extensively drug-resistant (XDR) Neisseria gonorrhoeae infection combining ceftriaxone-resistance and high-level azithromycin resistance, France, November 2022 and May 2023. *Euro Surveill* 2023;28(37) doi: 10.2807/1560-7917.ES.2023.28.37.2300456
13. Ouk V, Pham CD, Wi T, et al. The Enhanced Gonococcal Surveillance Programme, Cambodia. *Lancet Infect Dis* 2023;23(9):e332-e33. doi: 10.1016/S1473-3099(23)00479-6 [published Online First: 20230804]
14. Pleininger S, Indra A, Golparian D, et al. Extensively drug-resistant (XDR) Neisseria gonorrhoeae causing possible gonorrhoea treatment failure with ceftriaxone plus azithromycin in Austria, April 2022. *Euro Surveill* 2022;27(24) doi: 10.2807/1560-7917.ES.2022.27.24.2200455

For peer review only

15. Day M, Pitt R, Mody N, et al. Detection of 10 cases of ceftriaxone-resistant Neisseria gonorrhoeae in the United Kingdom, December 2021 to June 2022. *Euro Surveill* 2022;27(46) doi: 10.2807/1560-7917.ES.2022.27.46.2200803
16. Jennison AV, Whiley D, Lahra MM, et al. Genetic relatedness of ceftriaxone-resistant and high-level azithromycin resistant Neisseria gonorrhoeae cases, United Kingdom and Australia, February to April 2018. *Euro Surveill* 2019;24(8) doi: 10.2807/1560-7917.ES.2019.24.8.1900118
17. WHO. Global priority list of antibiotic-resistant bacteria to guide research, discovery, and development of new antibiotics 2017 [Available from: http://www.who.int/medicines/publications/global-priority-list-antibiotic-resistant-bacteria/en/ accessed 8 April 2018.
18. CDC. Antibiotic Resistance Threats in the United States, 2013 [Available from: http://www.cdc.gov/drugresistance/threat-report-2013/pdf/ar-threats-2013-508.pdf accessed 8 April 2018.
19. Department of Health. Responding to the threat of antimicrobial resistance: Australia's First National Antimicrobial Resistance Strategy 2015–2019. Canberra: Department of Health, Australian Government, 2015.
20. Petousis-Harris H, Paynter J, Morgan J, et al. Effectiveness of a group B outer membrane vesicle meningococcal vaccine against gonorrhoea in New Zealand: a retrospective case-control study. *Lancet* 2017;390(10102):1603-10. doi: 10.1016/S0140-6736(17)31449-6
21. Wang B, Giles L, Andraweera P, et al. Effectiveness and impact of the 4CMenB vaccine against invasive serogroup B meningococcal disease and gonorrhoea in an infant, child, and adolescent programme: an observational cohort and case-control study. *Lancet Infect Dis* 2022 doi: 10.1016/S1473-3099(21)00754-4 [published Online First: 2022/04/16]
22. Abara WE, Bernstein KT, Lewis FMT, et al. Effectiveness of a serogroup B outer membrane vesicle meningococcal vaccine against gonorrhoea: a retrospective observational study. *Lancet Infect Dis* 2022;22(7):1021-29. doi: 10.1016/S1473-3099(21)00812-4 [published Online First: 2022/04/16]
23. Robison SG, Leman RF. Association of Group B Meningococcal Vaccine Receipt With Reduced Gonorrhea Incidence Among University Students. *JAMA Netw Open* 2023;6(8):e2331742. doi: 10.1001/jamanetworkopen.2023.31742 [published Online First: 20230801]
24. Klee SR, Nassif X, Kusecek B, et al. Molecular and biological analysis of eight genetic islands that distinguish Neisseria meningitidis from the closely related pathogen Neisseria gonorrhoeae. *Infect Immun* 2000;68(4):2082-95. [published Online First: 2000/03/18]
25. Toneatto D, Pizza M, Masignani V, et al. Emerging experience with meningococcal serogroup B protein vaccines. *Expert review of vaccines* 2017;16(5):433-51. doi: 10.1080/14760584.2017.1308828
26. Semchenko EA, Tan A, Borrow R, et al. The Serogroup B Meningococcal Vaccine Bexsero Elicits Antibodies to Neisseria gonorrhoeae. *Clin Infect Dis* 2019;69(7):1101-11. doi: 10.1093/cid/ciy1061 [published Online First: 2018/12/15]
27. Wright E, Grulich A, Roy K, et al. Australasian Society for HIV, Viral Hepatitis and Sexual Health Medicine HIV pre-exposure prophylaxis: clinical guidelines. Update April 2018. *J Virus Erad* 2018;4(3):143-59. [published Online First: 2018/07/28]
28. GSK. Australian Product Informatio Bexsero (multicomponent meningococcal group b vaccine) suspension for injection. *https://wwwebstgagovau/ebs/picmi/picmirepositorynsf/pdf?OpenAgent&id=CP-2013-PI-02131-1&d=201904301016933* 2018
29. ASHM. Australian STI Management Guidelines For Use In Primary Care. *Australasian Society for HIV, Viral Hepatitis and Sexual Health Medicine (ASHM)* 2023;https://sti.guidelines.org.au/

For peer review only

For peer review only - http://bmjopen.bmj.com/site/about/guidelines.xhtml

30. Semchenko EA, Day CJ, Seib KL. MetQ of Neisseria gonorrhoeae Is a Surface-Expressed Antigen That Elicits Bactericidal and Functional Blocking Antibodies. *Infect Immun* 2017;85(2) doi: 10.1128/iai.00898-16 [published Online First: 2016/11/30]
31. Semchenko EA, Day CJ, Seib KL. The Neisseria gonorrhoeae Vaccine Candidate NHBA Elicits Antibodies That Are Bactericidal, Opsonophagocytic and That Reduce Gonococcal Adherence to Epithelial Cells. *Vaccines (Basel)* 2020;8(2) doi: 10.3390/vaccines8020219 [published Online First: 2020/05/18]
32. Bell SM, Pham JN, Rafferty DL, et al. Antibiotic susceptibility testing by the CDS method: a manual for medical and veterinary laboratories. 9th ed. *Kogarah, NSW: South Eastern Area Laboratory Services,* 2018;http://cdstest.net
33. Lahra MM, Hogan TR, Armstrong BH. Australian Gonococcal Surveillance Programme Annual Report, 2021. *Commun Dis Intell (2018)* 2022;46 doi: 10.33321/cdi.2022.46.52 [published Online First: 20220818]
34. Jolley KA, Bray JE, Maiden MCJ. Open-access bacterial population genomics: BIGSdb software, the PubMLST.org website and their applications. *Wellcome Open Res* 2018;3:124. doi: 10.12688/wellcomeopenres.14826.1 [published Online First: 20180924]
35. Williamson DA, Chow EPF, Gorrie CL, et al. Bridging of Neisseria gonorrhoeae lineages across sexual networks in the HIV pre-exposure prophylaxis era. *Nat Commun* 2019;10(1):3988. doi: 10.1038/s41467-019-12053-4 [published Online First: 2019/09/07]
36. Marshall GS, Abbing-Karahagopian V, Marshall HS, et al. A comprehensive review of clinical and real-world safety data for the four-component serogroup B meningococcal vaccine (4CMenB). *Expert review of vaccines* 2023;22(1):530-44. doi: 10.1080/14760584.2023.2222015
37. Callander D, Moreira C, El-Hayek C, et al. Monitoring the Control of Sexually Transmissible Infections and Blood-Borne Viruses: Protocol for the Australian Collaboration for Coordinated Enhanced Sentinel Surveillance (ACCESS). *JMIR Res Protoc* 2018;7(11):e11028. doi: 10.2196/11028 [published Online First: 2018/11/22]
38. World Health Organization (WHO). Global Health Sector Strategies on, respectively, HIV, viral hepatitis and sexually transmitted infections for the period 2022-2030, 2022.
39. Hui BB, Padeniya TN, Rebuli N, et al. A gonococcal vaccine has the potential to rapidly reduce the incidence of Neisseria gonorrhoeae infection among urban men who have sex with men. *J Infect Dis* 2022;225:983. doi: 10.1093/infdis/jiab581 [published Online First: 2021/12/12]
40. Bissessor M, Tabrizi SN, Fairley CK, et al. Differing Neisseria gonorrhoeae bacterial loads in the pharynx and rectum in men who have sex with men: implications for gonococcal detection, transmission, and control. *Journal of clinical microbiology* 2011;49(12):4304-06. doi: http://doi.org/10.1128/JCM.05341-11 [published Online First: 2011/09/28]
41. Cohen MS, Cannon JG, Jerse AE, et al. Human Experimentation with Neisseria gonorrhoeae: Rationale, Methods, and Implications for the Biology of Infection and Vaccine Development. *The Journal of Infectious Diseases* 1994;169(3):532-37. doi: http://doi.org/10.1093/infdis/169.3.532
42. Stupiansky NW, Van Der Pol B, Williams JA, et al. The Natural History of Incident Gonococcal Infection in Adolescent Women. *Sexually Transmitted Diseases* 2011;38(8) doi: http://doi.org/10.1097/OLQ.0b013e31820ff9a4
43. Moran JS, Levine WC. Drugs of choice for the treatment of uncomplicated gonococcal infections. *Clinical Infectious Diseases* 1995;20(Supplement_1):S47-S65. doi: https://doi.org/10.1093/clinids/20.Supplement_1.S47
44. Weinstock H, Workowski KA. Pharyngeal gonorrhea: An important reservoir of infection? *Clinical Infectious Diseases* 2009;49(12):1798-800. doi: https://doi.org/10.1086/648428
45. Read RC, Baxter D, Chadwick DR, et al. Effect of a quadrivalent meningococcal ACWY glycoconjugate or a serogroup B meningococcal vaccine on meningococcal carriage: an observer-blind, phase 3 randomised clinical trial. *Lancet* 2014 doi: 10.1016/S0140-6736(14)60842-4

For peer review only

46. McNamara LA, Thomas JD, MacNeil J, et al. Meningococcal Carriage Following a Vaccination Campaign With MenB-4C and MenB-FHbp in Response to a University Serogroup B Meningococcal Disease Outbreak-Oregon, 2015-2016. *J Infect Dis* 2017;216(9):1130-40. doi: 10.1093/infdis/jix446
47. Marshall HS, McMillan M, Koehler AP, et al. Meningococcal B Vaccine and Meningococcal Carriage in Adolescents in Australia. *N Engl J Med* 2020;382(4):318-27. doi: 10.1056/NEJMoa1900236
48. Gottlieb SL, Ndowa F, Hook EW, 3rd, et al. Gonococcal vaccines: Public health value and preferred product characteristics; report of a WHO global stakeholder consultation, January 2019. *Vaccine* 2020;38(28):4362-73. doi: 10.1016/j.vaccine.2020.02.073 [published Online First: 20200428]
49. Semchenko EA, Seib KL. Intractable problems require novel solutions: it's time to get serious about developing a gonorrhoea vaccine. *Sex Transm Infect* 2016;92(8):561-62. doi: 10.1136/sextrans-2015-052378 [published Online First: 2016/04/03]

For peer review only

For peer review only - http://bmjopen.bmj.com/site/about/guidelines.xhtml

Box 1 Study Inclusion and Exclusion Criteria

Inclusion criteria:

1. Between 18 to ≤ 50 years of age
2. Men (cis and trans), trans women and non-binary people who have had sex with at least one man in the last 6 months
3. Diagnosis of gonorrhoea or infectious syphilis in the last 18 months
4. Committed not to take doxycycline as prophylaxis for the duration of the trial
5. Able to understand spoken and written English
6. Willing and likely to comply with the trial procedures for 2 years
7. Agree to be contacted via SMS/phone/ email by the study team

   and either

   a. HIV-negative (with an HIV negative antibody test within 4 months of screening) and taking HIV PrEP (daily PrEP or on-demand PrEP) within the last 4 months at the time of enrolment or taking HIV post exposure prophylaxis (PEP) and there is a plan to start PrEP after the cessation of PEP, or
   b. HIV-positive and on an antiviral regimen, with an undetectable virus level of <200 copies/ml and a CD4 count >350 cells/cmm (to optimise the immune response to vaccine) within 12 months of screening

Exclusion criteria:

1. Have a previous history of vaccination for meningococcal B with 4CMenB
2. Have contraindications to receiving the meningococcal B vaccine which include:
   - Anaphylaxis following a previous dose of any meningococcal vaccine
   - Anaphylaxis following any vaccine component
3. Are participating in biomedical prevention strategies for bacterial STIs (participation in diagnostic or treatment studies is not an exclusion)
4. Are taking long-term (> 4 weeks) antibiotic for prophylaxis or treatment for acne, malaria, syphilis, or other bacterial condition(s)
5. Have defects in, or deficiency of, complement components, including factor H, factor D or properdin deficiency
6. Are taking or will receive complement inhibitors such as eculizumab (a monoclonal antibody directed against complement component C5) or ravulizumab
7. Have functional or anatomical asplenia, including sickle cell disease or other haemoglobinopathies, and congenital or acquired asplenia
8. Have had a haematopoietic stem cell transplant
9. Have any major unstable medical condition or therapy that may cause immune compromise (e.g., chemotherapy, radiation, corticosteroids [prednisone >5mg/day] within 14 days prior to screening)
10. Documented allergy to latex and/or kanamycin
11. Have prior known meningococcal disease
12. Positive pregnancy test at screening

For peer review only - http://bmjopen.bmj.com/site/about/guidelines.xhtml

Table 1 Primary, secondary and immunogenicity objectives and outcome measures of the GoGoVax study.

Objective*	Outcome measures
**Primary**	
To investigate whether the 4CMenB vaccine, when administered in a 2-dose regimen at 0 and 3 months, reduces the incidence of the first episode of *N. gonorrhoeae* infection, as determined by NAAT testing of urine samples, or swabs taken from the urethra, anorectum, oropharynx, and vagina, in GBM+ populations regardless of whether the infection is symptomatic or asymptomatic.	Detection of *N. gonorrhoeae* infection in the first instance in a urine sample or on a swab taken from the urethra, anorectum, oropharynx, or vagina, as determined by NAAT testing, post Month 4.
**Secondary**	
To investigate the impact of administration of a 2-dose regimen of 4CMenB vaccine on the incidence of the first episode of symptomatic *N. gonorrhoeae* infection of the urethra, anorectum or vagina, as determined by NAAT testing.	Detection of *N. gonorrhoeae* infection in the first instance in the urethra, anorectum, or vagina, as determined by NAAT testing, post Month 4, at a study visit when a participant also reports any symptoms at the relevant anatomic site.
To investigate the impact of a 2-dose regimen of 4CMenB vaccine on the incidence of the first episode of asymptomatic *N. gonorrhoeae* infection of the urethra, anorectum, oropharynx, or vagina, as determined by NAAT testing.	Detection of *N. gonorrhoeae* infection in the first instance in the urethra, anorectum, oropharynx, or vagina, as determined by NAAT testing, post Month 4, at a study visit when a participant reports no symptoms at the relevant anatomic site.
To investigate the impact of administration of a 2-dose regimen of 4CMenB vaccine on the incidence of first episode of *N. gonorrhoeae* infection, as determined by NAAT testing, regardless of symptoms and anatomic sites, by various *N. gonorrhoeae* strain types (genotype and AMR phenotype).	Detection of *N. gonorrhoeae* infection in the first instance in the urethra, anorectum, oropharynx, or vagina, as determined by NAAT testing post Month 4, by genotype and AMR phenotypes.
**Immunogenicity**	
To evaluate if the *N. gonorrhoeae*-specific enzyme-linked immunosorbent assay (ELISA), serum bactericidal activity assay (SBA) and/or opsonophagocytic killing assay (OPK) titres increase following 4CMenB vaccination.	The ELISA, SBA and OPK titres of serum and ELISA titres of oral mucosal transudates post 4CMenB dose 2, relative to baseline.
To evaluate if the *N. gonorrhoeae*-specific ELISA, SBA and/or OPK titres correlate with reduced *N. gonorrhoeae* infection.	The ELISA, SBA and OPK titres of serum during the study period.

*For all primary and secondary efficacy objectives, additional analyses will be conducted to allow repeated diagnoses of *N. gonorrhoeae* infection, as determined by NAAT testing from the same individuals to be included. This is to compare the overall incidence of all episodes of *N. gonorrhoeae* infection diagnosed during the study period between the vaccine and placebo arms, allowing multiple diagnoses of *N. gonorrhoeae* infection occurred in the same individuals at different time points.

For peer review only

Table 2. Summary of study visits

Study visit	Screening[a] (SCR)	Baseline (Month 0)	Month 3 (±14 days)	Month 6 (±14 days)	Month 9 (±14 days)	Month 12, 15, 18, 21 (±14 days)	Final visit Month 24 (±14 days)	Unscheduled[b]
**Study assessments**								
Informed consent	X							
Review eligibility	X		X					
Risk assessment – gonorrhoea or syphilis in the last 18 months	X							
Relevant medical history[c], demographics	X							
Concomitant antibiotic use, meningococcal B vaccination	X		X	X	X	X	X	X
Symptoms of urethritis, proctitis, epididymitis, and cervicitis/vaginitis	X		X	X	X	X	X	X
Pre-vaccination checklist		X	X					
Vaccine or placebo administration		X	X					
Serious adverse events review		X	X	X	X			
Study questionnaire		X				X[d]	X	
Randomisation[e]		X						
**Routine laboratory assessments**								
HIV antibody test[f]	X		X	X	X	X	X	
HIV viral load[s], CD4 count[g]	X							
STI tests[h] – urine, swabs & blood collection	X[i]		X	X	X	X	X	X
Pregnancy test[j]	X		X					
**Research specimens for central laboratory**								
Blood specimen (at selected study sites) – two 8ml samples (for Neisseria-specific immune response)		X[k]		X		X[l]	X	
Oral mucosal transudates (at selected study sites, for Neisseria-specific immune response)		X[k]		X		X[l]	X	
Culture isolates and NAAT samples (gonorrhoea infection incident cases only)	X		X	X	X	X	X	X

[a] All screening assessments are to be completed within 14 days of baseline (the visit when the first dose of 4CMenB or matched placebo will be administered).

[b] Unscheduled visit – an unscheduled visit will be conducted for 1) when a participant is diagnosed with symptomatic gonorrhoea infection by a NAAT test, or 2) when a participant is diagnosed with asymptomatic gonorrhoea infection by a NAAT test, or 3) when a participant returns for test of cure and has a positive gonorrhoea NAAT test.

[c] Including vaccination history for 4CMenB vaccine.

[d] Study questionnaire – Month 12 only.

[e] Randomisation can occur anytime between screening and baseline visit when the required HIV test result(s) and drug kit (containing the 4CMenB vaccine or matched placebo) are available, and an individual is confirmed to be eligible for the trial. Randomisation can occur on the same day of screening if the above aforementioned criteria are met.

[f] HIV negative participants only. An HIV negative antibody test within 4 months of screening can be used for randomisation if the participant has been taking PrEP (daily PrEP or on-demand PrEP) consistently. An individual starting PEP who has tested HIV negative within 4 months can be enrolled without waiting for the HIV antibody test result taken at

For peer review only

PEP initiation. In the rare occurrence that the HIV tests taken that day returns positive, and the participant has already had their first vaccine or placebo dose, the participant should not receive the second vaccine or placebo dose until their HIV viral load is <200 copies/ml and their CD4 is >350 cells/cmm.

[g] HIV-positive participants only. An undetectable HIV viral load of <200 copies/ml and CD4 count of >350 cells/cmm can be used for randomisation if the tests were conducted within 12 months of screening.

[h] Three-monthly STI tests as per the Australasian Society for HIV, Viral Hepatitis and Sexual Health Medicine (ASHM), Australian STI Management Guidelines For Use In Primary Care, *ASHM*, http://www.sti.guidelines.org.au/

[I] STI tests conducted within 14 days of screening do not have to repeated and the results can be used for screening.

[j] Pregnancy test for trans men and non-binary people who were recorded female at birth.

[k] Research specimens at baseline to be taken before the vaccine or placebo is given.

[l] Month 12 only.

For peer review only

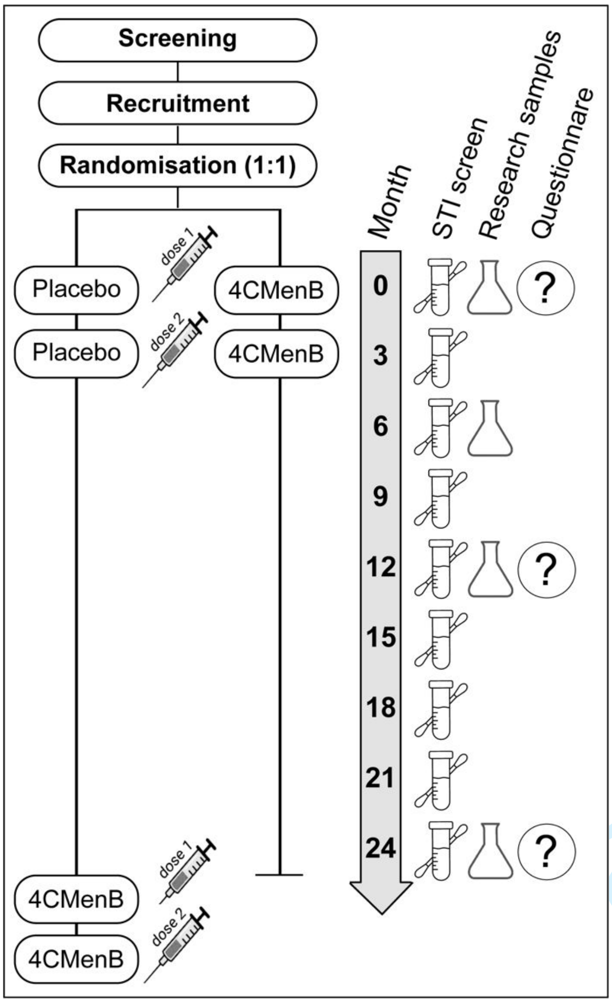

Figure 1. Study design of the GoGoVax trial.

For peer review only

SPIRIT

STANDARD PROTOCOL ITEMS: RECOMMENDATIONS FOR INTERVENTIONAL TRIALS

SPIRIT 2013 Checklist: Recommended items to address in a clinical trial protocol and related documents*

**Section/item**	**Item No**	**Description**	**Addressed on page number**
**Administrative information**			
Title	1	Descriptive title identifying the study design, population, interventions, and, if applicable, trial acronym	1
Trial registration	2a	Trial identifier and registry name. If not yet registered, name of intended registry	2
	2b	All items from the World Health Organization Trial Registration Data Set	Included in registry
Protocol version	3	Date and version identifier	8
Funding	4	Sources and types of financial, material, and other support	8
Roles and responsibilities	5a	Names, affiliations, and roles of protocol contributors	1
	5b	Name and contact information for the trial sponsor	1,8
	5c	Role of study sponsor and funders, if any, in study design; collection, management, analysis, and interpretation of data; writing of the report; and the decision to submit the report for publication, including whether they will have ultimate authority over any of these activities	8
	5d	Composition, roles, and responsibilities of the coordinating centre, steering committee, endpoint adjudication committee, data management team, and other individuals or groups overseeing the trial, if applicable (see Item 21a for data monitoring committee)	8

For peer review only

**Introduction**			
Background and rationale	6a	Description of research question and justification for undertaking the trial, including summary of relevant studies (published and unpublished) examining benefits and harms for each intervention	3
	6b	Explanation for choice of comparators	4
Objectives	7	Specific objectives or hypotheses	3, Table 1
Trial design	8	Description of trial design including type of trial (eg, parallel group, crossover, factorial, single group), allocation ratio, and framework (eg, superiority, equivalence, noninferiority, exploratory)	4
**Methods: Participants, interventions, and outcomes**			
Study setting	9	Description of study settings (eg, community clinic, academic hospital) and list of countries where data will be collected. Reference to where list of study sites can be obtained	4
Eligibility criteria	10	Inclusion and exclusion criteria for participants. If applicable, eligibility criteria for study centres and individuals who will perform the interventions (eg, surgeons, psychotherapists)	4, Box 1
Interventions	11a	Interventions for each group with sufficient detail to allow replication, including how and when they will be administered	4
	11b	Criteria for discontinuing or modifying allocated interventions for a given trial participant (eg, drug dose change in response to harms, participant request, or improving/worsening disease)	NA
	11c	Strategies to improve adherence to intervention protocols, and any procedures for monitoring adherence (eg, drug tablet return, laboratory tests)	NA
	11d	Relevant concomitant care and interventions that are permitted or prohibited during the trial	5
Outcomes	12	Primary, secondary, and other outcomes, including the specific measurement variable (eg, systolic blood pressure), analysis metric (eg, change from baseline, final value, time to event), method of aggregation (eg, median, proportion), and time point for each outcome. Explanation of the clinical relevance of chosen efficacy and harm outcomes is strongly recommended	5, Table 1

For peer review only - http://bmjopen.bmj.com/site/about/guidelines.xhtml

Participant timeline	13	Time schedule of enrolment, interventions (including any run-ins and washouts), assessments, and visits for participants. A schematic diagram is highly recommended (see Figure)	Table 2, Fig 1
Sample size	14	Estimated number of participants needed to achieve study objectives and how it was determined, including clinical and statistical assumptions supporting any sample size calculations	6
Recruitment	15	Strategies for achieving adequate participant enrolment to reach target sample size	4
**Methods: Assignment of interventions (for controlled trials)**			
Allocation:			
Sequence generation	16a	Method of generating the allocation sequence (eg, computer-generated random numbers), and list of any factors for stratification. To reduce predictability of a random sequence, details of any planned restriction (eg, blocking) should be provided in a separate document that is unavailable to those who enrol participants or assign interventions	4
Allocation concealment mechanism	16b	Mechanism of implementing the allocation sequence (eg, central telephone; sequentially numbered, opaque, sealed envelopes), describing any steps to conceal the sequence until interventions are assigned	4
Implementation	16c	Who will generate the allocation sequence, who will enrol participants, and who will assign participants to interventions	4
Blinding (masking)	17a	Who will be blinded after assignment to interventions (eg, trial participants, care providers, outcome assessors, data analysts), and how	4
	17b	If blinded, circumstances under which unblinding is permissible, and procedure for revealing a participant's allocated intervention during the trial	4
**Methods: Data collection, management, and analysis**			
Data collection methods	18a	Plans for assessment and collection of outcome, baseline, and other trial data, including any related processes to promote data quality (eg, duplicate measurements, training of assessors) and a description of study instruments (eg, questionnaires, laboratory tests) along with their reliability and validity, if known. Reference to where data collection forms can be found, if not in the protocol	5

For peer review only - http://bmjopen.bmj.com/site/about/guidelines.xhtml

	18b	Plans to promote participant retention and complete follow-up, including list of any outcome data to be collected for participants who discontinue or deviate from intervention protocols	5
Data management	19	Plans for data entry, coding, security, and storage, including any related processes to promote data quality (eg, double data entry; range checks for data values). Reference to where details of data management procedures can be found, if not in the protocol	5
Statistical methods	20a	Statistical methods for analysing primary and secondary outcomes. Reference to where other details of the statistical analysis plan can be found, if not in the protocol	6
	20b	Methods for any additional analyses (eg, subgroup and adjusted analyses)	6
	20c	Definition of analysis population relating to protocol non-adherence (eg, as randomised analysis), and any statistical methods to handle missing data (eg, multiple imputation)	6
**Methods: Monitoring**			
Data monitoring	21a	Composition of data monitoring committee (DMC); summary of its role and reporting structure; statement of whether it is independent from the sponsor and competing interests; and reference to where further details about its charter can be found, if not in the protocol. Alternatively, an explanation of why a DMC is not needed	5
	21b	Description of any interim analyses and stopping guidelines, including who will have access to these interim results and make the final decision to terminate the trial	NA
Harms	22	Plans for collecting, assessing, reporting, and managing solicited and spontaneously reported adverse events and other unintended effects of trial interventions or trial conduct	5
Auditing	23	Frequency and procedures for auditing trial conduct, if any, and whether the process will be independent from investigators and the sponsor	5
**Ethics and dissemination**			
Research ethics approval	24	Plans for seeking research ethics committee/institutional review board (REC/IRB) approval	2,7

For peer review only - http://bmjopen.bmj.com/site/about/guidelines.xhtml

Protocol amendments	25	Plans for communicating important protocol modifications (eg, changes to eligibility criteria, outcomes, analyses) to relevant parties (eg, investigators, REC/IRBs, trial participants, trial registries, journals, regulators)	NA
Consent or assent	26a	Who will obtain informed consent or assent from potential trial participants or authorised surrogates, and how (see Item 32)	4
	26b	Additional consent provisions for collection and use of participant data and biological specimens in ancillary studies, if applicable	NA
Confidentiality	27	How personal information about potential and enrolled participants will be collected, shared, and maintained in order to protect confidentiality before, during, and after the trial	5
Declaration of interests	28	Financial and other competing interests for principal investigators for the overall trial and each study site	8
Access to data	29	Statement of who will have access to the final trial dataset, and disclosure of contractual agreements that limit such access for investigators	6
Ancillary and post-trial care	30	Provisions, if any, for ancillary and post-trial care, and for compensation to those who suffer harm from trial participation	5
Dissemination policy	31a	Plans for investigators and sponsor to communicate trial results to participants, healthcare professionals, the public, and other relevant groups (eg, via publication, reporting in results databases, or other data sharing arrangements), including any publication restrictions	2,8
	31b	Authorship eligibility guidelines and any intended use of professional writers	8,9
	31c	Plans, if any, for granting public access to the full protocol, participant-level dataset, and statistical code	NA
**Appendices**			
Informed consent materials	32	Model consent form and other related documentation given to participants and authorised surrogates	
Biological specimens	33	Plans for collection, laboratory evaluation, and storage of biological specimens for genetic or molecular analysis in the current trial and for future use in ancillary studies, if applicable	5

For peer review only - http://bmjopen.bmj.com/site/about/guidelines.xhtml

*It is strongly recommended that this checklist be read in conjunction with the SPIRIT 2013 Explanation & Elaboration for important clarification on the items. Amendments to the protocol should be tracked and dated. The SPIRIT checklist is copyrighted by the SPIRIT Group under the Creative Commons "Attribution-NonCommercial-NoDerivs 3.0 Unported" license.

For peer review only

BMJ Open

A multicentre double-blind randomised placebo-controlled trial evaluating the efficacy of the meningococcal B vaccine, 4CMenB (Bexsero), against Neisseria gonorrhoeae infection in men who have sex with men: the GoGoVax study Protocol

Journal:	*BMJ Open*
Manuscript ID	bmjopen-2023-081675.R1
Article Type:	Protocol
Date Submitted by the Author:	29-Feb-2024

Complete List of Authors:	Seib , KL; Griffith University Institute for Glycomics, ; Griffith University<br>Donovan, Basil; University of New South Wales, The Kirby Institute<br>Thng, Caroline; Queensland Health, Gold Coast Sexual Health<br>Lewis, David ; Western Sydney Sexual Health Centre<br>McNulty, Anna; Sydney Sexual Health Centre,<br>Fairley, Christopher; Melbourne Sexual Health Centre,<br>Yeung, Barbara; The Kirby Institute, UNSW<br>Jin, Fengyi; The Kirby Institute, UNSW,<br>Fraser, Doug; The Kirby Institute, UNSW<br>Bavinton , BR; The Kirby Institute<br>Law, Matthew; University of New South Wales,<br>Chen, Marcus; Melbourne Sexual Health Centre, The Alfred Hospital<br>Chow, Eric; University of Melbourne; Melbourne Sexual Health Centre<br>Whiley, David; The University of Queensland, Queensland Children's Medical Research Institute<br>Mackie, Brent; ACON<br>Jennings, Michael; Griffith University, Institute for Glycomics<br>Jennison, Amy; Public Health Microbiology, Queensland Health Forensic and Scientific Services,<br>Lahra, Monica M; New South Wales Health Pathology<br>Grulich, Andrew; The Kirby Institute, ;

**Primary Subject Heading**:	Infectious diseases
Secondary Subject Heading:	Infectious diseases, Sexual health, Immunology (including allergy)
Keywords:	Vaccination, Clinical Trial, IMMUNOLOGY, MICROBIOLOGY, Sexually Transmitted Disease

SCHOLARONE™
Manuscripts

A multicentre double-blind randomised placebo-controlled trial evaluating the efficacy of the meningococcal B vaccine, 4CMenB (Bexsero), against *Neisseria gonorrhoeae* infection in men who have sex with men: the GoGoVax study Protocol

Kate L Seib [1*], Basil Donovan [2], Caroline Thng [3], David A Lewis [4,5], Anna McNulty [6,7], Christopher K Fairley [8,9], Barbara Yeung [2], Fengyi Jin [2], Doug Fraser [2], Benjamin R Bavinton [2], Matthew Law [2], Marcus Y Chen [8,9], Eric PF Chow [8,9], David M Whiley [10], Brent Mackie [11], Michael P Jennings [1], Amy V Jennison [12], Monica M Lahra [13], Andrew E Grulich [2].

[1] Institute for Glycomics, Griffith University, Gold Coast, QLD, Australia
[2] The Kirby Institute, University of New South Wales, Sydney, NSW, Australia
[3] Gold Coast Sexual Health, Southport, QLD, Australia
[4] Western Sydney Sexual Health Centre, Sydney, NSW, Australia
[5] Sydney Medical School-Westmead, Faculty of Medicine and Health and Sydney Infectious Diseases Institute, University of Sydney, Sydney, NSW, Australiasdfdfdfd
[6] Sydney Sexual Health Centre, Sydney, NSW, Australia
[7] School of Population Health, UNSW Sydney, Sydney, Australia.
[8] Melbourne Sexual Health Centre, Melbourne, VIC, Australia
[9] Central Clinical School, Faculty of Medicine, Nursing and Health Sciences, Monash University, Melbourne, VIC, Australia
[10] UQ Centre for Clinical Research, Faculty of Medicine, The University of Queensland, Brisbane, QLD, Australia
[11] ACON Health, NSW, Australia
[12] Public Health Microbiology, Queensland Health Forensic and Scientific Services, Brisbane, QLD, Australia
[13] WHO Collaborating Centre for STI and AMR, NSW Health Pathology Microbiology. The Prince of Wales Hospital, Sydney, NSW, Australia

*Correspondence: Kate L Seib, k.seib@griffith.edu.au

For peer review only

For peer review only - http://bmjopen.bmj.com/site/about/guidelines.xhtml

ABSTRACT

Introduction Gonorrhoea, the sexually transmissible infection caused by *Neisseria gonorrhoeae*, has a substantial impact on sexual and reproductive health globally with an estimated 82 million new infections each year worldwide. *N. gonorrhoeae* antimicrobial resistance continues to escalate, and disease control is largely reliant on effective therapy as there is no proven effective gonococcal vaccine available. However, there is increasing evidence from observational cohort studies that the serogroup B meningococcal vaccine 4CMenB (trade name Bexsero®), licenced to prevent invasive disease caused by *Neisseria meningitidis*, may provide cross-protection against the closely related bacterium *N. gonorrhoeae.* This study will evaluate the efficacy of 4CMenB against *N. gonorrhoeae* infection in men (cis and trans), trans women and non-binary people who have sex with men (hereafter referred to as GBM+).

Methods and analysis This is a double-blind, randomised placebo-controlled trial in GBM+, either HIV-negative on pre-exposure prophylaxis (PrEP) against HIV or living with HIV (CD4 count >350 cells/mm^3), who have had a diagnosis of gonorrhoea or infectious syphilis in the last 18 months (a key characteristic associated with a high risk of *N. gonorrhoeae* infection). Participants are randomised 1:1 to receive two doses of 4CMenB or placebo 3 months apart. Participants have 3-monthly visits over 24 months, which include testing for *N. gonorrhoeae* and other sexually transmissible infections, collection of demographics, sexual behaviour risks and antibiotic use, and collection of research samples for analysis of *N. gonorrhoeae*-specific systemic and mucosal immune responses. The primary outcome is the incidence of the first episode of *N. gonorrhoeae* infection, as determined by nucleic acid amplification tests (NAATs), post month 4. Additional outcomes consider the incidence of symptomatic or asymptomatic *N. gonorrhoeae* infection at different anatomical sites (i.e., urogenital, anorectum, or oropharynx), incidence by *N. gonorrhoeae* genotype and antimicrobial resistance phenotype, and level and functional activity of *N. gonorrhoeae*-specific antibodies.

Ethics and dissemination Ethical approval was obtained from the St Vincent's Hospital Human Research Ethics Committee (HREC), St Vincent's Hospital Sydney, NSW, Australia (ref: 2020/ETH01084). Results will be disseminated in peer-reviewed journals and via presentation at national and international conferences.

Trial registration ClinicalTrials.gov Identifier NCT04415424.

Keywords

Gonorrhoea, *Neisseria gonorrhoeae,* sexually transmissible infection (STI), men who have sex with men (MSM), gay and bisexual men (GBM), meningococcal vaccine, 4CMenB, Bexsero®.

Strengths and limitations of this study

- This is the first double-blind randomised placebo-controlled trial to evaluate the efficacy of the four-component meningitis B vaccine (4CMenB) against *N. gonorrhoeae* infection in men who have sex with men and will provide information regarding vaccine efficacy against symptomatic and asymptomatic infection at different anatomic sites.
- Antimicrobial resistance (AMR) testing and genome sequencing of *N. gonorrhoeae* positive samples will provide information regarding the circulating strains in the study population, and the vaccine efficacy against circulating strains including those with differing AMR profiles.
- Immunological analysis of samples from the trial will provide insight into the *N. gonorrhoeae* specific immune response following 4CMenB vaccination.
- Limitations of the study are that it does not include cisgender women, immunosuppressed individuals living with HIV (CD4 count ≤350 cells/mm^3), or participants with a low risk of *N. gonorrhoeae* infection.

For peer review only - http://bmjopen.bmj.com/site/about/guidelines.xhtml

INTRODUCTION

Gonorrhoea is a global public health concern due to its prevalence, the severe sequelae it can cause if left untreated, and the increasing difficulty in treating multi-drug resistant strains of *Neisseria gonorrhoeae*.(1 2) There are estimated to be more than 82 million new cases of *N. gonorrhoeae* infections annually worldwide,(3) and the incidence is rising. Between 2012 and 2019 there was a 127% increase in notified gonorrhoea cases in Australia,(4) and a 74% increase in the USA.(5) In Australia, the majority of gonococcal infections are in are in gay, bisexual and other men who have sex with men (GBM).(6) An Australian state-wide implementation project of pre-exposure prophylaxis (PrEP) against HIV infection in GBM documented a gonorrhoea incidence of 39.0 per 100-person years (PY), with infected sites including the urethra (7.3/100 PY), pharynx (19.7/100 PY), and anus (22.6/100 PY).(7) Analysis from sexual health clinics across Australia indicated that HIV-positive GBM had a gonorrhoea incidence of 29.1/100 PY.(8)

The outcomes of *N. gonorrhoeae* infection vary by site of infection and by sex.(1) Oropharyngeal and anorectal infections are typically asymptomatic. Symptomatic gonococcal infection typically presents as urethritis in males and cervicitis in females, although up to 10% of male and ~30-80% of female genital tract infections are asymptomatic.(3) Undiagnosed or untreated urogenital tract infections can lead to severe sequelae, including urethral stricture, urogenital tract abscesses, epididymo-orchitis and infertility in males, and pelvic inflammatory disease, adverse pregnancy outcomes and infertility in females.(3) *N. gonorrhoeae* occasionally disseminates, resulting in sepsis, septic arthritis, perihepatitis, endocarditis and/or meningitis. Infection with *N. gonorrhoeae* may also increase the risk of acquiring and transmitting HIV.(1)

N. gonorrhoeae AMR is of utmost concern, particularly with emerging resistance to ceftriaxone. In 2018 extensively drug-resistant (and ceftriaxone-resistant) *N. gonorrhoeae* were identified in Australia, resistant to all commonly used antibiotics, and very recently further isolates were reported in the UK, Austria, Cambodia, and France.(9-16) As such, *N. gonorrhoeae* has been prioritised as an urgent public health threat for which new preventive measures are needed, such as a vaccine.(17-19) Unfortunately, gonococcal vaccine development has been unsuccessful to date and there are large knowledge gaps regarding what constitutes a protective immune response for *N. gonorrhoeae.*(1) However, there is increasing evidence from observational cohort and case-control studies that outer membrane vesicle (OMV)-based *Neisseria meningitidis* (meningococcal) serogroup B (MenB) vaccines may provide cross-protection against gonorrhoea.(20-23) *N. gonorrhoeae* and *N. meningitidis* are closely related bacteria, with conservation of the majority of genes (24) and thus also conservation of outer membrane proteins that could be targeted by vaccines. Observational studies have detected a decrease in incidence of *N. gonorrhoeae* infection following vaccination with the outer membrane vesicle (OMV) meningococcal vaccines MeNZB (20) or 4CMenB (21-23) with vaccine effectiveness estimated to be approximately 30-50%. The OMVs in these vaccines are structures that are released from the outer membrane of Gram-negative bacteria, and contain phospholipids, lipooligosaccharide and a complex mix of proteins. The broader spectrum four-component serogroup B vaccine, 4CMenB (marketed as Bexsero®) contains the MeNZB OMV antigens plus an additional three recombinant antigens (NHBA-GNA1030 and fHbp-GNA2091 fusions, and NadA).(25) There are homologues of 20 of the 22 major OMV vaccine proteins present in *N gonorrhoeae*, as well as the NHBA antigen.(26) Therefore, there are several vaccine antigens that may be responsible for inducing an immune response that cross reacts with *N. gonorrhoeae.* The observational study findings with MenB OMV-based vaccines are the first time that any vaccine has been associated with protection against gonorrhoea. However, data from a randomised control trial is required to confirm the efficacy of 4CMenB to protect against *N. gonorrhoeae* infection.

STUDY AIMS AND OBJECTIVES

This study aims to evaluate the efficacy of 4CMenB in the prevention of *N. gonorrhoeae* infection in men (cis and trans), trans women, trans men, and non-binary people who have sex with men (hereafter referred to as GBM+). The primary outcome is the incidence of the first episode of *N. gonorrhoeae*

For peer review only - http://bmjopen.bmj.com/site/about/guidelines.xhtml

infection, as determined by nucleic acid amplification tests (NAATs), post month 4 (i.e., after 4 weeks post dose two). Secondary outcomes are the incidence of symptomatic or asymptomatic *N. gonorrhoeae* infection at different anatomical sites (i.e., urogenital, anorectum, or oropharynx), incidence by *N. gonorrhoeae* genotype and antimicrobial resistance phenotype, and level and functional activity of any induced *N. gonorrhoeae*-specific antibodies. Primary, secondary and immunogenicity objectives and endpoint measures are outlined in Table 1.

METHODS AND ANALYSIS

Study design and setting

The study is a double-blind, randomised placebo-controlled trial of a two-dose schedule of 4CMenB to prevent urethral, oropharyngeal, and anal *N. gonorrhoeae* infection in GBM+, who are either taking HIV PrEP or postexposure prophylaxis (PEP), are planning to start PrEP after the cessation of PEP, or are living with HIV with a CD4 count >350 cells/mm^3. Participants are randomised 1:1 to receive two doses of 4CMenB or saline placebo 3 months apart. Participants are required to attend 3-monthly follow-up visits over 24 months. At each visit there is testing for *N. gonorrhoeae* and other sexually transmissible infections including HIV, collection of blood and oral mucosal exudate samples for analysis of *N. gonorrhoeae*-specific immune responses, and collection of demographics, sexual behaviour risks, and antibiotic use. An overview of the study design is shown in Figure 1 and a summary of the study visits is shown in Table 2.

The study is being conducted at five public sexual health clinics and two private clinics in New South Wales, Victoria and Queensland, Australia: the Sydney Sexual Health Centre (SSHC), the Western Sydney Sexual Health Centre (WSSHC), the Melbourne Sexual Health Centre (MSHC), the Gold Coast Sexual Health Service (GCSHS), Royal Prince Alfred Sexual Health (RPASH) Sydney, Taylor Square Private Clinic (TSPC) Sydney, and Prahran Market Clinic (PMC) Melbourne.

Study population, recruitment, and eligibility criteria

The participant population for this trial is GBM+ taking PrEP/PEP or living with HIV (CD4 count >350 cells/mm^3), who have had a diagnosis of gonorrhoea or infectious syphilis in the last 18 months (a key characteristic associated with greater gonorrhoea incidence(7)). This population is recommended under Australian guidelines to attend clinics 3-monthly for comprehensive sexual health screening, including NAAT-based screening of urogenital, oropharyngeal, and anal specimens for *N. gonorrhoeae* infection.(6 27)

Potentially eligible participants were identified by clinicians at each clinic based on the inclusion and exclusion criteria (see Box 1) and were referred to the study clinicians or study nurses who explained the study, checked eligibility, and obtained informed consent. Potentially eligible participants were also referred to the participating clinics by other public sexual health centres, public health clinics, general practices, and state-based lesbian, gay, bisexual, and transgender community organisations, such as ACON and Positive Life NSW in NSW, Thorne Harbour Health and Living Positive Victoria in Victoria, and the Queensland Council for LGBTI Health and Queensland Positive People.

Study processes

Randomisation and blinding

Once recruited, participants were randomly assigned (1:1) and stratified by clinical sites via a web-based randomisation system, to receive either 4CMenB or saline placebo. Participants, their clinicians (site investigators/doctors, site coordinators/nurses) and study researchers assessing the outcomes were blinded to the assignment. The site pharmacists and drug manager who dispensed the study treatment, and the vaccinators who administered the study treatment were unblinded to the treatment allocation.

Intervention

A pre-vaccination checklist was completed prior to administration of intervention to ensure safety of

For peer review only - http://bmjopen.bmj.com/site/about/guidelines.xhtml

participants. At the month 0 (Baseline) and month 3 visits participants received either a dose of 0.5 ml 4CMenB or a dose of 0.5 ml placebo (150 mmol of sodium chloride / 0.9% saline solution) by intramuscular injection. This vaccine schedule is consistent with 4CMenB's indication for immunisation against invasive disease caused by *N. meningitidis* group B in people >2 years of age (two doses with an interval of ≥1 month).(28) 4CMenB is a licensed vaccine in Australia with well documented safety profile but it is not approved by the Therapeutic Good Administration (TGA) for the prevention of gonorrhoea. Therefore, the trial is being conducted under the Clinical Trial Notification (CTN) scheme. At the completion of study, participants who were randomised to the placebo arm will be given the opportunity to receive the 4CMenB vaccine.

Sample and data collection

The study assessments, samples, and data collected at each 3-monthly visits over 24 months are outlined in Figure 1 and Table 2. At each 3-monthly visit, study participants have routine specimens collected as part of their PrEP or HIV clinical care as per the Australian STI Management Guidelines.(29) These specimens include urogenital, oropharyngeal and anorectal specimens for screening for *N. gonorrhoeae* and *Chlamydia trachomatis* by nucleic acid amplification test (NAAT), and blood for screening for HIV and syphilis. At each visit, data are also recorded regarding symptoms that may be related to gonorrhoea including urethritis, proctitis, epididymitis, and cervicitis/vaginitis. Unscheduled visits to the participating sites or external healthcare providers, at which gonorrhoea is diagnosed by a NAAT, are also recorded.

Participants complete a study questionnaire at baseline to collect demographics and sexual risk behaviour. A questionnaire is also completed at months 12 and 24 to collect ongoing sexual risk behaviour. At each visit, recent antibiotic use, and history of meningococcal B vaccination within the last 3 months is also recorded.

Research specimens (blood and oral mucosal transudates) are collected at baseline, and at Months 0, 6, 12, and 24 at selected study sites to investigate vaccine-induced antibodies via assays including enzyme-linked immunosorbent assays (ELISA), serum bactericidal activity (SBA), opsonophagocytic killing (OPA), and antigen function blocking assays.(26 30 31). Culture isolates collected as part of the routine care for participants with *N. gonorrhoeae* infection will be used to conduct phenotypic antimicrobial resistance testing (32 33) and whole genome sequence analysis.(34 35) Testing will be performed at the WHO Collaborating Centre for STIs and AMR at the Prince of Wales Hospital in Sydney, the Queensland Forensic and Scientific Services at Queensland Health in Brisbane, and/or at the Microbiological Diagnostic Unit in Melbourne.

Safety reporting

4CMenB is a licensed vaccine with a well-documented safety and tolerability profile.(36) Therefore, safety monitoring is focused on serious adverse events (SAEs). All SAEs will be reported within 24 hours to the Study Sponsor and the Project Medical Officer (at the Kirby Institute) and to the HREC annually. The Sponsor will notify the TGA, HREC and site principal investigators of all significant events that can adversely affect the safety of participants.

Data management

Source documents include, but are not limited to participant medical records, laboratory reports, participant progress notes, pharmacy records and any other reports or records of procedures performed in accordance with the protocol. Study data are collected using the Medrio Electronic Data Capture system (Medrio Inc., San Francisco, CA, US). Medrio is a cloud-based, web-enabled password protected platform. Each participant is assigned a unique Participant Identification Number (PIN) that is documented in the participant's medical record and all study documents including the dispensing records. Data are stored using Google Cloud Platform facilities globally. Medrio is adherent to ICH GCP, FDA 21 CFR Part 11; EudraLex, Annex 11; GDPR; and HIPAA. All site data

For peer review only - http://bmjopen.bmj.com/site/about/guidelines.xhtml

entry personnel will be required to pass training and assessments to be able to enter data and all will have their own confidential login credentials. The login password will be changed every 3 months. Following each participant visit the designated site staff complete the visit-specific electronic case report form (eCRF) as soon as possible after the completion of the visit. There will be no personal information or full identifiable information of any participants entered or stored in the study database. Participants also provide consent for the Project Team to acquire any other information and results from other health services for the purpose of the research via linkage with the Australian Collaboration for Coordinated Enhanced Sentinel Surveillance (ACCESS) system.(37) Data from ACCESS will enable long-term follow-up after the completion of the study even if a participant moves to another clinic. At The Kirby Institute, UNSW Sydney (Sponsor), designated research personnel have viewing access to the EDC to monitor data and conduct source data verification. The study data will be extracted after the database is closed at the end of the study. The study data will be kept for 15 years after study completion.

Sample size

The primary endpoint of the study will be incidence of first episode of *N. gonorrhoeae* infection (urogenital, anal, or oropharyngeal) as determined by NAAT testing, per 100 PY, post month 4. Assuming a vaccine efficacy of 30% (hazard ratio=0.7; as predicted for MeNZB™),(20) and a conservative *N. gonorrhoeae* incidence of 25 per 100 PY (based on recent Australian data for symptomatic and asymptomatic infections in GBM on PrEP (39 per 100 PY)(7) and in HIV-positive GBM (29 per 100 PY)(8)), 1:1 randomisation, a 12-month recruitment period, and 24-month follow-up, and 10% lost to follow-up, a total of 730 participants (365 randomised to each arm) would need to be recruited to have 80% power to demonstrate vaccine efficacy (2-alpha=5%).

Statistical analysis

The primary efficacy endpoint is the incidence of first episode of *N. gonorrhoeae* infection detected by a NAAT, post month 4. A secondary endpoint will include the incidence of all episodes of *N. gonorrhoeae* infection detected by a NAAT, including those within 4 weeks post vaccine dose 2. All episodes of chlamydial infection diagnosed during the study period will also be captured for the proposed additional analyses to compare the incidence of chlamydial infection between the vaccine arm and the placebo arm.

Analysis populations

The primary analysis of efficacy will be per-protocol, including study participants who received two vaccine or placebo doses, have two or more follow-up visits following the second dose, and do not fail on any exclusion criterion pertinent to the assessment of efficacy. In this analysis, only *N. gonorrhoeae* infections 4 weeks post dose 2 (i.e., after Month 4) will be considered. A modified intent-to-treat (MITT) analysis will be performed as a secondary analysis including all participants (including general protocol violators) who receive at least 1 dose of vaccine or placebo and have at least 6 months of study follow-up after the first dose. For these analyses, any efficacy against cases occurring after Day 1 visit will be counted.

Statistical Methods

Vaccine efficacy (VE) is expressed as a percentage and will be defined as:

$VE = 100\{1- (r_v/r_p)\}$

where r_v is the incidence rate among vaccine recipients and r_p is the incidence rate among placebo recipients. Each efficacy hypothesis will be tested by constructing a two-sided 95% confidence interval for VE using Poisson regression models, accommodating repeated measures for the secondary endpoint.

Patient and public involvement

For peer review only - http://bmjopen.bmj.com/site/about/guidelines.xhtml

Representatives from the community groups ACON (an LGBTQ+ health organisation) and Positive Life NSW (a community organisation for people living with HIV) have been involved in the research since the initial funding application and have participated in monthly/2-monthly management team meetings since the start of trial planning. They provided input on the protocol, study information and recruitment materials. These community partners will assist in dissemination of study results to participants and the community via their social media platforms.

DISCUSSION

In light of increasing gonorrhoea incidence and escalating antimicrobial resistance, there is an urgent need for new gonorrhoea prevention strategies. The World Health Organization (WHO) has set a target for reducing gonorrhoea incidence by 90% by 2030 and highlighted the need for an effective vaccine to help achieve this goal.(38) GoGoVax is the first double-blind, randomised placebo-controlled trial to investigate the efficacy of the meningococcal 4CMenB vaccine against *N. gonorrhoeae* infection in GBM+. This trial will provide critical scientific evidence to inform potential indication expansion of 4CMenB to include gonorrhoea, which could lead to a substantial reduction in *N. gonorrhoeae* incidence worldwide. Observational evidence to date suggests that 4CMenB may have a vaccine effectiveness against *N. gonorrhoeae* of approximately 30-50%.(21-23) Mathematical modelling in an MSM population indicates that a gonococcal vaccine with 25-50% efficacy and 2-years duration of protection could reduce gonorrhoea prevalence by 31-62% in 2 years if 30% of MSM presenting for STI screening are vaccinated per visit.(39)

Our study addresses several questions in the field regarding overall protection against *N. gonorrhoeae* infection provided by 4CMenB, but also regarding vaccine efficacy against symptomatic and asymptomatic infection at genital and extragenital sites of infection, efficacy against diverse strains, as well as duration and potential mechanisms of vaccine-mediated protection. The link between symptoms and *N. gonorrhoeae* load and transmission is unclear (40-42) and it is unknown if vaccine efficacy will differ depending on the presence or absence of symptoms. Vaccine efficacy may also differ by anatomical site of infection. This has been seen for antibiotic treatment of gonorrhoea, with antibiotics being less effective against oropharyngeal gonorrhoea than for urethral and rectal infections.(43 44) Although 4CMenB is able to prevent invasive meningococcal disease, it is not believed to result in a clinically significant reduction in nasopharyngeal carriage of *N. meningitidis*.(45-47) However, unlike *N. meningitidis*, *N. gonorrhoeae* does not have a polysaccharide capsule which may alter its susceptibility to 4CMenB vaccine-induced immune responses in the pharynx. Given the diversity of *N. gonorrhoeae*, the antimicrobial resistance testing and genome sequencing of *N. gonorrhoeae* positive samples will provide information regarding the circulating strains in the population to enable evaluation of vaccine efficacy against circulating strains, including of differing AMR profiles. Linkage between our study and the Australian ACCESS surveillance system(37) will allow assessment of durability of any preventive effect of 4CMenB beyond the duration of the trial. Immunological analysis of samples from the trial will provide insight into the *N. gonorrhoeae*-specific immune response following 4CMenB vaccination. Immunological correlates of protection for *N. gonorrhoeae* have not yet been identified,(48 49) and insight from this trial will facilitate future vaccine development and evaluation.

This trial is targeted at the people at highest risk. Men who have sex with men and people living with HIV are key populations at higher risk for gonococcal infection (48) and Australian GBM+ are diagnosed with some of the highest gonorrhoea incidence rates ever described.(7) (8) As such, it is essential to understand whether 4CMenB is able to prevent or reduce *N. gonorrhoeae* in this population. However, it should be noted that we did not enrol immunosuppressed men living with HIV (CD4 count <350 cells/mm^3) in order to optimise the immune response to vaccine in participants. Another important limitation of our study is the lack of inclusion of cisgender women. Gonorrhoea causes a significant burden of disease in females, and it is possible that past *N. gonorrhoeae* infection

For peer review only - http://bmjopen.bmj.com/site/about/guidelines.xhtml

may impact vaccine-induced immune responses. Additional 4CMenB efficacy trials currently underway in different settings that include female participants (e.g., clinicaltrials.gov NCT04350138) will hopefully address some of these limitations and complement findings from our study.

Due for completion in 2025, the GoGoVax study will inform future gonorrhoea prevention strategies. If 4CMenB is proven to be effective against *N. gonorrhoeae*, this could facilitate changes in gonorrhoea prevention strategies that could improve sexual and reproductive health worldwide.

ETHICS AND DISSEMINATION

The current study protocol (version 5, 24 June 2022) informed consent forms, and all recruitment materials have been reviewed and approved by St Vincent's Hospital Human Research Ethics Committee, St Vincent's Hospital Sydney, NSW, Australia (ref: 2020/ETH01084). The study results will be published in international peer-reviewed journals, presented at national and international conferences, and provided to study participants and the public via social, television, radio and/or print media.

Acknowledgements

We would like to acknowledge the GoGoVax Protocol Steering Committee members and the site investigators and coordinators.

Protocol Steering Committee members:

Prof Kate Seib, Prof Basil Donovan, Scientia Prof Andrew Grulich, Prof Christopher Fairley, Prof David Lewis, A/Prof Anna McNulty, Dr Caroline Thng, A/Prof Mark Polizzotto, A/Prof David Whiley, Prof Matthew Law, A/Prof Eric Chow, Dr Rob Finlayson, Dr Mark O'Reilly, Dr Nick Comninos, Dr Rick Varma, Dr Norman Roth, Dr Beng Eu, Prof David Templeton, Dr Amy Jennison, Mr Brent Mackie, Mr Andrew Heslop, Ms Jane Costello, Dr Christine Selvey, Prof Deborah Williamson, Prof John Kaldor, A/Prof Marcus Chen, Prof Michael Jennings, Prof Monica Lahra, Prof Rebecca Guy, Dr Fengyi Jin, Dr Benjamin Bavinton, Ms Barbara Yeung, Mr Doug Fraser, Ms. Michelle Burns.

Site investigators and coordinators:

Sydney Sexual Health Centre - A/Prof Anna McNulty, Dr Rick Varma, Mr. Paul Robinson, Mr. Anik Ray, Ms. Alison Jenkins.

Western Sydney Sexual Health Centre - Prof David Lewis, Dr Nick Comninos, Dr Rohan Bopage, Ms. Melissa Power, Mr. Pradeep Kumar.

Melbourne Sexual Health Centre - Prof Christopher Fairley, A/Prof Eric Chow, A/Prof Marcus Chen, Ms. Kate Maddaford, Mr. Finn Mercury.

Gold Coast Sexual Health Services - Dr Caroline Thng, Ms. Catherine Donald, Helen McEvoy.

RPA Sexual Health - Prof David Templeton, Dr David Atefi, Dr Rachel Burdon, Dr Lara Whitbourne, Ms. Linda Garton, Ms. Ashlee Jackson.

Taylor Square Private Clinic - Dr Rob Finlayson, Dr Mark O'Reilly, Ms. Piper Calleia, Mr. Kiran Muthukrishnan.

Prahran Market Clinic - Dr Norman Roth, Dr Beng Eu, Ms. Helen Lau.

Contributors

KLS, BD and AEG designed the study and wrote the initial manuscript. KLS, BD, CT, DAL, AM, CKF, BY, FJ, DF, BRB, ML, MYC, EPFC, DMW, BM, MPJ, AVJ, MML, AEG contributed to study design, protocol development, and manuscript review, and approved the final version of the manuscript for submission.

Funding

For peer review only - http://bmjopen.bmj.com/site/about/guidelines.xhtml

This work was supported by the Australian National and Medical Health Council (NHMRC) grant number 1182443. Additional support was provided by Griffith University (Administrative Institute) and the Kirby Institute UNSW Sydney (Study Sponsor). Vaccine was provided by GlaxoSmithKline (GSK) Australia Pty Ltd. The funding bodies, study sponsor and GSK had no role in the design of the study. KLS acknowledges supported from the NHMRC (grants 2017383 and 2002182). EPFC is supported by an NHMRC Emerging Leadership Investigator Grant (GNT1172873).

Competing interests
None

Ethics approval St Vincent's Hospital Human Research Ethics Committee (HREC/2020/ETH01084).

Patient consent for publication Not required.
Provenance and peer review Not commissioned; externally peer reviewed.

References

1. Edwards JL, Jennings MP, Apicella MA, et al. Is gonococcal disease preventable? The importance of understanding immunity and pathogenesis in vaccine development. *Crit Rev Microbiol* 2016;42(6):928-41. doi: 10.3109/1040841X.2015.1105782 [published Online First: 2016/01/26]
2. Unemo M, Bradshaw CS, Hocking JS, et al. Sexually transmitted infections: challenges ahead. *The Lancet Infectious diseases* 2017;17(8):e235-e79. doi: 10.1016/S1473-3099(17)30310-9 [published Online First: 2017/07/14]
3. WHO. Global incidence and prevalence of selected curable sexually transmitted infections – 2008 2012 [Available from: http://www.who.int/reproductivehealth/publications/rtis/stisestimates/en/ accessed 7 March 2015.
4. Kirby Institute. HIV, viral hepatitis and sexually transmissible infections in Australia: annual surveillance report 2018. The Kirby Institute, UNSW Sydney, 2018.
5. CDC. Sexually Transmitted Disease Surveillance 2017. *https://wwwcdcgov/std/stats* 2018
6. ASHM. The Australian STI Management Guidelines. *http://wwwstiguidelinesorgau/* 2019
7. Traeger MW, Cornelisse VJ, Asselin J, et al. Association of HIV Preexposure Prophylaxis With Incidence of Sexually Transmitted Infections Among Individuals at High Risk of HIV Infection. *JAMA* 2019;321(14):1380-90. doi: 10.1001/jama.2019.2947 [published Online First: 2019/04/10]
8. Callander D, Guy R, Fairley CK, et al. Gonorrhoea gone wild: rising incidence of gonorrhoea and associated risk factors among gay and bisexual men attending Australian sexual health clinics. *Sex Health* 2019;16(5):457-63. doi: 10.1071/SH18097 [published Online First: 2018/11/10]
9. Eyre DW, Sanderson ND, Lord E, et al. Gonorrhoea treatment failure caused by a Neisseria gonorrhoeae strain with combined ceftriaxone and high-level azithromycin resistance, England, February 2018. *Euro Surveill* 2018;23(27) doi: 10.2807/1560-7917.ES.2018.23.27.1800323 [published Online First: 2018/07/12]
10. ECDC. Extensively drug-resistant (XDR) Neisseria gonorrhoeae in the United Kingdom and Australia. *European Centre for Disease Prevention and Control Stockholm* *https://ecdceuropaeu* 2018
11. Whiley DM, Jennison A, Pearson J, et al. Genetic characterisation of Neisseria gonorrhoeae resistant to both ceftriaxone and azithromycin. *Lancet Infect Dis* 2018;18(7):717-18. doi: 10.1016/S1473-3099(18)30340-2 [published Online First: 2018/07/07]

For peer review only - http://bmjopen.bmj.com/site/about/guidelines.xhtml

12. Maubaret C, Camelena F, Mrimeche M, et al. Two cases of extensively drug-resistant (XDR) Neisseria gonorrhoeae infection combining ceftriaxone-resistance and high-level azithromycin resistance, France, November 2022 and May 2023. *Euro Surveill* 2023;28(37) doi: 10.2807/1560-7917.ES.2023.28.37.2300456
13. Ouk V, Pham CD, Wi T, et al. The Enhanced Gonococcal Surveillance Programme, Cambodia. *Lancet Infect Dis* 2023;23(9):e332-e33. doi: 10.1016/S1473-3099(23)00479-6 [published Online First: 20230804]
14. Pleininger S, Indra A, Golparian D, et al. Extensively drug-resistant (XDR) Neisseria gonorrhoeae causing possible gonorrhoea treatment failure with ceftriaxone plus azithromycin in Austria, April 2022. *Euro Surveill* 2022;27(24) doi: 10.2807/1560-7917.ES.2022.27.24.2200455
15. Day M, Pitt R, Mody N, et al. Detection of 10 cases of ceftriaxone-resistant Neisseria gonorrhoeae in the United Kingdom, December 2021 to June 2022. *Euro Surveill* 2022;27(46) doi: 10.2807/1560-7917.ES.2022.27.46.2200803
16. Jennison AV, Whiley D, Lahra MM, et al. Genetic relatedness of ceftriaxone-resistant and high-level azithromycin resistant Neisseria gonorrhoeae cases, United Kingdom and Australia, February to April 2018. *Euro Surveill* 2019;24(8) doi: 10.2807/1560-7917.ES.2019.24.8.1900118
17. WHO. Global priority list of antibiotic-resistant bacteria to guide research, discovery, and development of new antibiotics 2017 [Available from: http://www.who.int/medicines/publications/global-priority-list-antibiotic-resistant-bacteria/en/ accessed 8 April 2018.
18. CDC. Antibiotic Resistance Threats in the United States, 2013 [Available from: http://www.cdc.gov/drugresistance/threat-report-2013/pdf/ar-threats-2013-508.pdf accessed 8 April 2018.
19. Department of Health. Responding to the threat of antimicrobial resistance: Australia's First National Antimicrobial Resistance Strategy 2015–2019. Canberra: Department of Health, Australian Government, 2015.
20. Petousis-Harris H, Paynter J, Morgan J, et al. Effectiveness of a group B outer membrane vesicle meningococcal vaccine against gonorrhoea in New Zealand: a retrospective case-control study. *Lancet* 2017;390(10102):1603-10. doi: 10.1016/S0140-6736(17)31449-6
21. Wang B, Giles L, Andraweera P, et al. Effectiveness and impact of the 4CMenB vaccine against invasive serogroup B meningococcal disease and gonorrhoea in an infant, child, and adolescent programme: an observational cohort and case-control study. *Lancet Infect Dis* 2022 doi: 10.1016/S1473-3099(21)00754-4 [published Online First: 2022/04/16]
22. Abara WE, Bernstein KT, Lewis FMT, et al. Effectiveness of a serogroup B outer membrane vesicle meningococcal vaccine against gonorrhoea: a retrospective observational study. *Lancet Infect Dis* 2022;22(7):1021-29. doi: 10.1016/S1473-3099(21)00812-4 [published Online First: 2022/04/16]
23. Robison SG, Leman RF. Association of Group B Meningococcal Vaccine Receipt With Reduced Gonorrhea Incidence Among University Students. *JAMA Netw Open* 2023;6(8):e2331742. doi: 10.1001/jamanetworkopen.2023.31742 [published Online First: 20230801]
24. Klee SR, Nassif X, Kusecek B, et al. Molecular and biological analysis of eight genetic islands that distinguish Neisseria meningitidis from the closely related pathogen Neisseria gonorrhoeae. *Infect Immun* 2000;68(4):2082-95. [published Online First: 2000/03/18]
25. Toneatto D, Pizza M, Masignani V, et al. Emerging experience with meningococcal serogroup B protein vaccines. *Expert review of vaccines* 2017;16(5):433-51. doi: 10.1080/14760584.2017.1308828
26. Semchenko EA, Tan A, Borrow R, et al. The Serogroup B Meningococcal Vaccine Bexsero Elicits Antibodies to Neisseria gonorrhoeae. *Clin Infect Dis* 2019;69(7):1101-11. doi: 10.1093/cid/ciy1061 [published Online First: 2018/12/15]

For peer review only

27. Wright E, Grulich A, Roy K, et al. Australasian Society for HIV, Viral Hepatitis and Sexual Health Medicine HIV pre-exposure prophylaxis: clinical guidelines. Update April 2018. *J Virus Erad* 2018;4(3):143-59. [published Online First: 2018/07/28]
28. GSK. Australian Product Informatio Bexsero (multicomponent meningococcal group b vaccine) suspension for injection. *https://wwwebstgagovau/ebs/picmi/picmirepositorynsf/pdf?OpenAgent&id=CP-2013-PI-02131-1&d=201904301016933* 2018
29. ASHM. Australian STI Management Guidelines For Use In Primary Care. *Australasian Society for HIV, Viral Hepatitis and Sexual Health Medicine (ASHM)* 2023;https://sti.guidelines.org.au/
30. Semchenko EA, Day CJ, Seib KL. MetQ of Neisseria gonorrhoeae Is a Surface-Expressed Antigen That Elicits Bactericidal and Functional Blocking Antibodies. *Infect Immun* 2017;85(2) doi: 10.1128/iai.00898-16 [published Online First: 2016/11/30]
31. Semchenko EA, Day CJ, Seib KL. The Neisseria gonorrhoeae Vaccine Candidate NHBA Elicits Antibodies That Are Bactericidal, Opsonophagocytic and That Reduce Gonococcal Adherence to Epithelial Cells. *Vaccines (Basel)* 2020;8(2) doi: 10.3390/vaccines8020219 [published Online First: 2020/05/18]
32. Bell SM, Pham JN, Rafferty DL, et al. Antibiotic susceptibility testing by the CDS method: a manual for medical and veterinary laboratories. 9th ed. *Kogarah, NSW: South Eastern Area Laboratory Services,* 2018;http://cdstest.net
33. Lahra MM, Hogan TR, Armstrong BH. Australian Gonococcal Surveillance Programme Annual Report, 2021. *Commun Dis Intell (2018)* 2022;46 doi: 10.33321/cdi.2022.46.52 [published Online First: 20220818]
34. Jolley KA, Bray JE, Maiden MCJ. Open-access bacterial population genomics: BIGSdb software, the PubMLST.org website and their applications. *Wellcome Open Res* 2018;3:124. doi: 10.12688/wellcomeopenres.14826.1 [published Online First: 20180924]
35. Williamson DA, Chow EPF, Gorrie CL, et al. Bridging of Neisseria gonorrhoeae lineages across sexual networks in the HIV pre-exposure prophylaxis era. *Nat Commun* 2019;10(1):3988. doi: 10.1038/s41467-019-12053-4 [published Online First: 2019/09/07]
36. Marshall GS, Abbing-Karahagopian V, Marshall HS, et al. A comprehensive review of clinical and real-world safety data for the four-component serogroup B meningococcal vaccine (4CMenB). *Expert review of vaccines* 2023;22(1):530-44. doi: 10.1080/14760584.2023.2222015
37. Callander D, Moreira C, El-Hayek C, et al. Monitoring the Control of Sexually Transmissible Infections and Blood-Borne Viruses: Protocol for the Australian Collaboration for Coordinated Enhanced Sentinel Surveillance (ACCESS). *JMIR Res Protoc* 2018;7(11):e11028. doi: 10.2196/11028 [published Online First: 2018/11/22]
38. World Health Organization (WHO). Global Health Sector Strategies on, respectively, HIV, viral hepatitis and sexually transmitted infections for the period 2022-2030, 2022.
39. Hui BB, Padeniya TN, Rebuli N, et al. A gonococcal vaccine has the potential to rapidly reduce the incidence of Neisseria gonorrhoeae infection among urban men who have sex with men. *J Infect Dis* 2022;225:983. doi: 10.1093/infdis/jiab581 [published Online First: 2021/12/12]
40. Bissessor M, Tabrizi SN, Fairley CK, et al. Differing Neisseria gonorrhoeae bacterial loads in the pharynx and rectum in men who have sex with men: implications for gonococcal detection, transmission, and control. *Journal of clinical microbiology* 2011;49(12):4304-06. doi: http://doi.org/10.1128/JCM.05341-11 [published Online First: 2011/09/28]
41. Cohen MS, Cannon JG, Jerse AE, et al. Human Experimentation with Neisseria gonorrhoeae: Rationale, Methods, and Implications for the Biology of Infection and Vaccine Development. *The Journal of Infectious Diseases* 1994;169(3):532-37. doi: http://doi.org/10.1093/infdis/169.3.532

For peer review only

42. Stupiansky NW, Van Der Pol B, Williams JA, et al. The Natural History of Incident Gonococcal Infection in Adolescent Women. *Sexually Transmitted Diseases* 2011;38(8) doi: http://doi.org/10.1097/OLQ.0b013e31820ff9a4
43. Moran JS, Levine WC. Drugs of choice for the treatment of uncomplicated gonococcal infections. *Clinical Infectious Diseases* 1995;20(Supplement_1):S47-S65. doi: https://doi.org/10.1093/clinids/20.Supplement_1.S47
44. Weinstock H, Workowski KA. Pharyngeal gonorrhea: An important reservoir of infection? *Clinical Infectious Diseases* 2009;49(12):1798-800. doi: https://doi.org/10.1086/648428
45. Read RC, Baxter D, Chadwick DR, et al. Effect of a quadrivalent meningococcal ACWY glycoconjugate or a serogroup B meningococcal vaccine on meningococcal carriage: an observer-blind, phase 3 randomised clinical trial. *Lancet* 2014 doi: 10.1016/S0140-6736(14)60842-4
46. McNamara LA, Thomas JD, MacNeil J, et al. Meningococcal Carriage Following a Vaccination Campaign With MenB-4C and MenB-FHbp in Response to a University Serogroup B Meningococcal Disease Outbreak-Oregon, 2015-2016. *J Infect Dis* 2017;216(9):1130-40. doi: 10.1093/infdis/jix446
47. Marshall HS, McMillan M, Koehler AP, et al. Meningococcal B Vaccine and Meningococcal Carriage in Adolescents in Australia. *N Engl J Med* 2020;382(4):318-27. doi: 10.1056/NEJMoa1900236
48. Gottlieb SL, Ndowa F, Hook EW, 3rd, et al. Gonococcal vaccines: Public health value and preferred product characteristics; report of a WHO global stakeholder consultation, January 2019. *Vaccine* 2020;38(28):4362-73. doi: 10.1016/j.vaccine.2020.02.073 [published Online First: 20200428]
49. Semchenko EA, Seib KL. Intractable problems require novel solutions: it's time to get serious about developing a gonorrhoea vaccine. *Sex Transm Infect* 2016;92(8):561-62. doi: 10.1136/sextrans-2015-052378 [published Online First: 2016/04/03]

For peer review only

Box 1 Study Inclusion and Exclusion Criteria

Inclusion criteria:

1. Between 18 to ≤ 50 years of age
2. Men (cis and trans), trans women and non-binary people who have had sex with at least one man in the last 6 months
3. Diagnosis of gonorrhoea or infectious syphilis in the last 18 months
4. Committed not to take doxycycline as prophylaxis for the duration of the trial*
5. Able to understand spoken and written English
6. Willing and likely to comply with the trial procedures for 2 years
7. Agree to be contacted via SMS/phone/ email by the study team
   and either
   a. HIV-negative (with an HIV negative antibody test within 4 months of screening) and taking HIV PrEP (daily PrEP or on-demand PrEP) within the last 4 months at the time of enrolment or taking HIV post exposure prophylaxis (PEP) and there is a plan to start PrEP after the cessation of PEP, or
   b. HIV-positive and on an antiviral regimen, with an undetectable virus level of <200 copies/ml and a CD4 count >350 cells/cmm (to optimise the immune response to vaccine) within 12 months of screening

Exclusion criteria:

1. Have a previous history of vaccination for meningococcal B with 4CMenB
2. Have contraindications to receiving the meningococcal B vaccine which include:
   - Anaphylaxis following a previous dose of any meningococcal vaccine
   - Anaphylaxis following any vaccine component
3. Are participating in biomedical prevention strategies for bacterial STIs (participation in diagnostic or treatment studies is not an exclusion)
4. Are taking long-term (> 4 weeks) antibiotic for prophylaxis or treatment for acne, malaria, syphilis, or other bacterial condition(s)
5. Have defects in, or deficiency of, complement components, including factor H, factor D or properdin deficiency
6. Are taking or will receive complement inhibitors such as eculizumab (a monoclonal antibody directed against complement component C5) or ravulizumab
7. Have functional or anatomical asplenia, including sickle cell disease or other haemoglobinopathies, and congenital or acquired asplenia
8. Have had a haematopoietic stem cell transplant
9. Have any major unstable medical condition or therapy that may cause immune compromise (e.g., chemotherapy, radiation, corticosteroids [prednisone >5mg/day] within 14 days prior to screening)
10. Documented allergy to latex and/or kanamycin
11. Have prior known meningococcal disease
12. Positive pregnancy test at screening

* In accordance with the Australasian Society for HIV, Viral Hepatitis and Sexual Health Medicine (ASHM) 2023 Consensus Statement on doxycycline prophylaxis (Doxy-PEP) (https://ashm.org.au/about/news/doxy-pep-statement/), site clinicians were advised that they could prescribe doxycycline to study participants if they felt this was appropriate for their patients, and the number of days participants have taken doxycycline is recorded at each visit.

For peer review only - http://bmjopen.bmj.com/site/about/guidelines.xhtml

Table 1 Primary, secondary and immunogenicity objectives and outcome measures of the GoGoVax study.

**Objective***	**Outcome measures**
**Primary**	
To investigate whether the 4CMenB vaccine, when administered in a 2-dose regimen at 0 and 3 months, reduces the incidence of the first episode of *N. gonorrhoeae* infection, as determined by NAAT testing of urine samples, or swabs taken from the urethra, anorectum, oropharynx, and vagina, in GBM+ populations regardless of whether the infection is symptomatic or asymptomatic.	Detection of *N. gonorrhoeae* infection in the first instance in a urine sample or on a swab taken from the urethra, anorectum, oropharynx, or vagina, as determined by NAAT testing, post Month 4.
**Secondary**	
To investigate the impact of administration of a 2-dose regimen of 4CMenB vaccine on the incidence of the first episode of symptomatic *N. gonorrhoeae* infection of the urethra, anorectum or vagina, as determined by NAAT testing.	Detection of *N. gonorrhoeae* infection in the first instance in the urethra, anorectum, or vagina, as determined by NAAT testing, post Month 4, at a study visit when a participant also reports any symptoms at the relevant anatomic site.
To investigate the impact of a 2-dose regimen of 4CMenB vaccine on the incidence of the first episode of asymptomatic *N. gonorrhoeae* infection of the urethra, anorectum, oropharynx, or vagina, as determined by NAAT testing.	Detection of *N. gonorrhoeae* infection in the first instance in the urethra, anorectum, oropharynx, or vagina, as determined by NAAT testing, post Month 4, at a study visit when a participant reports no symptoms at the relevant anatomic site.
To investigate the impact of administration of a 2-dose regimen of 4CMenB vaccine on the incidence of first episode of *N. gonorrhoeae* infection, as determined by NAAT testing, regardless of symptoms and anatomic sites, by various *N. gonorrhoeae* strain types (genotype and AMR phenotype).	Detection of *N. gonorrhoeae* infection in the first instance in the urethra, anorectum, oropharynx, or vagina, as determined by NAAT testing post Month 4, by genotype and AMR phenotypes.
**Immunogenicity**	
To evaluate if the *N. gonorrhoeae*-specific enzyme-linked immunosorbent assay (ELISA), serum bactericidal activity assay (SBA) and/or opsonophagocytic killing assay (OPK) titres increase following 4CMenB vaccination.	The ELISA, SBA and OPK titres of serum and ELISA titres of oral mucosal transudates post 4CMenB dose 2, relative to baseline.
To evaluate if the *N. gonorrhoeae*-specific ELISA, SBA and/or OPK titres correlate with reduced *N. gonorrhoeae* infection.	The ELISA, SBA and OPK titres of serum during the study period.

*For all primary and secondary efficacy objectives, additional analyses will be conducted to allow repeated diagnoses of *N. gonorrhoeae* infection, as determined by NAAT testing from the same individuals to be included. This is to compare the overall incidence of all episodes of *N. gonorrhoeae* infection diagnosed during the study period between the vaccine and placebo arms, allowing multiple diagnoses of *N. gonorrhoeae* infection occurred in the same individuals at different time points.

For peer review only - http://bmjopen.bmj.com/site/about/guidelines.xhtml

Table 2. Summary of study visits

Study visit	Screening[a] (SCR)	Baseline (Month 0)	Month 3 (±14 days)	Month 6 (±14 days)	Month 9 (±14 days)	Month 12, 15, 18, 21 (±14 days)	Final visit Month 24 (±14 days)	Unscheduled[b]
**Study assessments**								
Informed consent	X							
Review eligibility	X		X					
Risk assessment – gonorrhoea or syphilis in the last 18 months	X							
Relevant medical history[c], demographics	X							
Concomitant antibiotic use, meningococcal B vaccination	X		X	X	X	X	X	X
Symptoms of urethritis, proctitis, epididymitis, and cervicitis/vaginitis	X		X	X	X	X	X	X
Pre-vaccination checklist		X	X					
Vaccine or placebo administration		X	X					
Serious adverse events review		X	X	X	X			
Study questionnaire		X				X[d]	X	
Randomisation[e]		X						
**Routine laboratory assessments**								
HIV antibody test[f]	X		X	X	X	X	X	
HIV viral load[s], CD4 count[g]	X							
STI tests[h] – urine, swabs & blood collection	X[i]		X	X	X	X	X	X
Pregnancy test[j]	X		X					
**Research specimens for central laboratory**								
Blood specimen (at selected study sites) – two 8ml samples (for Neisseria-specific immune response)		X[k]		X		X[l]	X	
Oral mucosal transudates (at selected study sites, for Neisseria-specific immune response)		X[k]		X		X[l]	X	
Culture isolates and NAAT samples (gonorrhoea infection incident cases only)	X		X	X	X	X	X	X

[a] All screening assessments are to be completed within 14 days of baseline (the visit when the first dose of 4CMenB or matched placebo will be administered). The reason participants failed screening or were ineligible to be randomised or take part in the study is recorded.

[b] Unscheduled visit – an unscheduled visit will be conducted for 1) when a participant is diagnosed with symptomatic gonorrhoea infection by a NAAT test, or 2) when a participant is diagnosed with asymptomatic gonorrhoea infection by a NAAT test, or 3) when a participant returns for test of cure and has a positive gonorrhoea NAAT test.

[c] Including vaccination history for 4CMenB vaccine.

[d] Study questionnaire – Month 12 only.

[e] Randomisation can occur anytime between screening and baseline visit when the required HIV test result(s) and drug kit (containing the 4CMenB vaccine or matched placebo) are available, and an individual is confirmed to be eligible for the trial. Randomisation can occur on the same day of screening if the above aforementioned criteria are met.

For peer review only - http://bmjopen.bmj.com/site/about/guidelines.xhtml

For peer review only

[f] HIV negative participants only. An HIV negative antibody test within 4 months of screening can be used for randomisation if the participant has been taking PrEP (daily PrEP or on-demand PrEP) consistently. An individual starting PEP who has tested HIV negative within 4 months can be enrolled without waiting for the HIV antibody test result taken at PEP initiation. In the rare occurrence that the HIV tests taken that day returns positive, and the participant has already had their first vaccine or placebo dose, the participant should not receive the second vaccine or placebo dose until their HIV viral load is <200 copies/ml and their CD4 is >350 cells/cmm.

[g] HIV-positive participants only. An undetectable HIV viral load of <200 copies/ml and CD4 count of >350 cells/cmm can be used for randomisation if the tests were conducted within 12 months of screening.

[h] Three-monthly STI tests as per the Australasian Society for HIV, Viral Hepatitis and Sexual Health Medicine (ASHM), Australian STI Management Guidelines For Use In Primary Care, *ASHM*, http://www.sti.guidelines.org.au/

[I] STI tests conducted within 14 days of screening do not have to repeated and the results can be used for screening.

[j] Pregnancy test for trans men and non-binary people who were recorded female at birth.

[k] Research specimens at baseline to be taken before the vaccine or placebo is given.

[l] Month 12 only.

For peer review only

655
656 **Figure 1. Study design of the GoGoVax trial.**
657

For peer review only

For peer review only - http://bmjopen.bmj.com/site/about/guidelines.xhtml

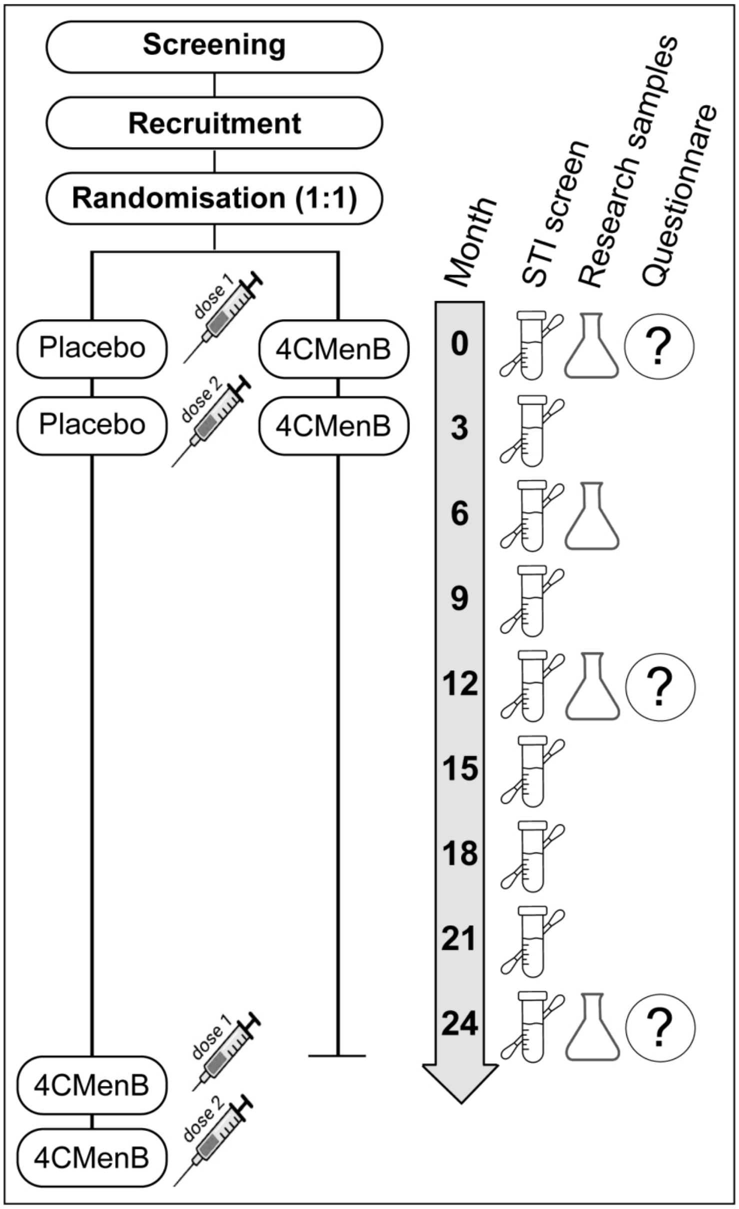

121x198mm (300 x 300 DPI)
